# Sequential combination of cisplatin with eugenol targets ovarian cancer stem cells through the Notch-Hes1 signalling pathway

**DOI:** 10.1186/s13046-019-1360-3

**Published:** 2019-08-30

**Authors:** Syed S. Islam, Abdelilah Aboussekhra

**Affiliations:** Cancer Biology and Experimental Therapeutics, Department of Molecular Oncology, King Faisal Specialist Hospital and Research Centre, Riyadh, Saudi Arabia

**Keywords:** Notch, Hes1, Cisplatin, Eugenol, Ovarian cancer stem cells

## Abstract

**Background:**

Ovarian carcinomas are the deadliest gynecological malignancies owing to their high rate of recurrence and high resistance to platinum-based chemotherapy. Recent studies have shown platinum-dependent enrichment of ovarian tumors with side population as well as cancer stem cells, which are highly resistant to the treatment. To overcome this treatment-limiting factor, we sought to combine cisplatin with eugenol, a natural substance known to have anti-cancer effects.

**Methods:**

The efficiency of combining cisplatin with eugenol was first tested in vitro on two ovarian cancer cell lines SKOV3 and OV2774 using the WST1 and the flow cytometry techniques. The effect of this combination on ovarian cancer stem cells was determined by the tumorsphere formation assay, while the implication of the Notch pathway was evaluated post-ectopic expression of the Hes1 gene. The resulting changes in the expression of several markers was assessed by immunoblotting, immunofluorescence as well as quantitative RT-PCR. Cell sorting was also used to isolate specific ovarian cancer sub-population of cells. Furthermore, tumor-bearing mouse models were utilized to prove the potential therapeutic value of the cisplatin/eugenol combination treatment in vivo.

**Results:**

We have shown that adding eugenol to cisplatin-treated ovarian cancer cells synergistically inhibited their growth and survival through induction of apoptosis. Importantly, this sequential inhibition strongly reduced the proportion of side population cells and suppressed cisplatin-dependent enrichment in ovarian cancer stem cells. Additionally, while increase in the level of Hes1 promoted stemness and enhanced resistance to cisplatin, cisplatin/eugenol cotreatment inhibited the Notch-Hes1 pathway and strongly downregulated the drug resistance ABC transporter genes. These findings were confirmed in vivo by showing that cisplatin/eugenol cotherapy inhibited tumor growth in animals, reduced the proportion and self-renewal capacities of cancer stem cells and significantly improved disease-free survival of tumor-bearing animals compared with either therapy alone.

**Conclusions:**

These results indicate that cisplatin/eugenol sequential combination could be of great therapeutic value for ovarian cancer patients through targeting the Notch-Hes1 pathway and the consequent elimination of the resistant cancer stem cells.

**Electronic supplementary material:**

The online version of this article (10.1186/s13046-019-1360-3) contains supplementary material, which is available to authorized users.

## Background

Ovarian cancer (OC) remains the deadliest gynecological malignancy in the world [1]. While most patients (70–80%) respond favourably to first-line treatment and achieve clinical remission, the majority of the patients experience resistance to therapy and recurrence [2, 3]. This poor clinical outcome of OC is thought to be due to the presence of high cellular heterogeneity and the existence of OC stem cells (OCSCs), a sub-fraction of cancer cells known to mediate resistance to chemotherapies [4]. Indeed, recurrent and metastatic OC are often resistant to platinum-based chemotherapy [5, 6]. Evidence suggests that cisplatin-dependent enrichment in OCSCs is primarily due to the activation of various stemness-related pathways, including the Notch signalling [7]. The alteration of the Notch pathway genes has been reported in approximately 22% of all analyzed ovarian tumors [8]. OC tumors express high level of the Notch3 mRNA and protein, which correlates with drug resistance and poor overall survival [9]. The Notch pathway is primarily initiated by binding of the Notch ligands Jagged and Delta to the Notch receptors. Once bound, it activates the Hes/Hey transcription family members and other downstream genes by releasing the intracellular domains of the Notch receptors (NICD) through proteolytic cleavage mediated by the gamma-secretase [10, 11]. Recent studies have pointed out that the Notch pathway plays crucial roles in the self-renewal, drug resistance, the promotion and maintenance of cancer stem cells (CSCs) and assist to escape the most cytotoxic therapies [7]. Therefore, targeting CSCs through inhibiting the Notch pathway could be of great therapeutic value.

We have recently shown that the natural dietary product, eugenol can potentiate the cisplatin effect against breast cancer cells through targeting the CSCs sub-population [12]. Eugenol is widely used in traditional medicine, primarily as an antiseptic, and anti-bacterial agent [13]. Analysis of source and purification of eugenol have been described previously [14]. Therefore, we have extended our present study to evaluate whether combination of eugenol and cisplatin may produce similar outcome against OC cells and OCSCs. We have shown that eugenol can suppress cisplatin-dependent promotion of OCSCs enrichment and stemness in OC cells through targeting the Notch pathway both in vitro and in vivo.

## Materials and methods

### Cell lines

SKOV3 and OV2774 (also known as MDAH-2774) cell lines were originally obtained from American Type Cell Culture (ATCC, Manassas, VA). Cells were grown in DMEM/F2 (1:1) (Gibco, MD, USA) containing 10% FBS and 1% antibiotics at 37 °C in a 5% CO2 incubator. Cells were regularly screened for mycoplasma contamination using MycoAlert Mycoplasma Detection Kits (Lonza).

### Animals

Five weeks female Nu/J mice were used for in vivo experiments. The animal study was approved by the King Faisal Specialist Hospital and Research Centre and the animal study protocols were approved by the animal use committee (protocol/RAC#2170 034). Animals were injected with 1 × 10^6^ cells/mouse into the dorsal flank (10/group) and allowed to develop tumors. Once palpable, tumors were identified, mice were given intramuscular injection daily with eugenol (cat # E51719; Sigma, MO, USA) (50 mg/Kg), cisplatin (cat # 1134357, Sigma, MO, USA) (2 mg/Kg) and combination of both drugs for 21 days. Tumor growth and mouse body weight was recorded every alternate day. Mice were sacrificed at day 22, tumor samples were collected, frozen immediately for histological analysis or dissociated tumor cells were used for further in vitro and in vivo assays.

### Cell growth, cytotoxicity and apoptosis assays

Please see Additional file 7: materials and methods for detailed description.

### Isolation of Hes1GFP (+) and GFP (−) cells by flow cytometry

OV2774 and SKOV3 cells were transfected by dHes1d2-driven EGFP reporter plasmid [15] and cultured in the DMEM/F12 (1:1) complete medium. Hes1GFP^+^ and GFP^−^ cells were sorted using FACSAria cell sorter (BD Bioscience, USA). Hes1GFP^+^ cells were maintained and expanded as spheres in an ultra-low attachment 6-well plates (BD Bioscience), supplemented with 2% B27 supplements (Invitrogen, Carlsbad, CA), 20 ng/ml of epidermal growth factor (EGF, Sigma, St Louis, MD), 20 ng/ml basic fibroblast growth factor (bFGF, Sigma), 50 ng/ml hydrocortisone (Sigma), 2 μg/ml insulin, 4 μg/ml heparin (Stem Cell Technologies, BC, Canada) for 20 days. List of antibodies for flow cytometry is in the Additional file 8: Table S1.

### Sphere assay

FACS sorted OV2774 and SKOV3 cells were cultured in 6-well ultra-low attachment plates at a density of 5000 viable cells/well, supplemented with 0.4% BSA, 1% penicillin and streptomycin, B27, 20 ng/ml hEGF, 5 μg/ml insulin, 20 ng/ml FGF, 50 ng/ml hydrocortisone and 4 μg/ml heparin. Spheres were treated with cisplatin, eugenol and the combination of both. The number and size of spheres were viewed under microscope and counted the number of spheres every 3 days. To assess the effects of the drugs on secondary and tertiary sphere formation, cells were grown in the absence of drugs.

### Statistical analysis

For statistical analysis and graphing we used the R-statistical software, (version 3.4.4). R packages: DRC for cell survival and cytotoxicity; ggplot2 for all graphing, CORRPLOT for correlation matrix, STATMOD for Extreme Limiting Dilution Assay (ELDA); ‘Survival’ and ‘survminer 0.3.0’ for tumor free survival analysis. All data presented in this study as the mean +/−SD. Triplicate results were analyzed by two-tailed Student *t test*. A *p* value of < 0.05 considered statistically significant. For multiple comparisons, 1 and 2-way analysis of variance (ANOVA) was performed using the R- Statistical software “ggplo2” package.

**Note:** Additional information is described in the Additional file 7: Materials and methods section.

## Results

### Eugenol sensitizes OC cells to cisplatin

**Fig. 1:**
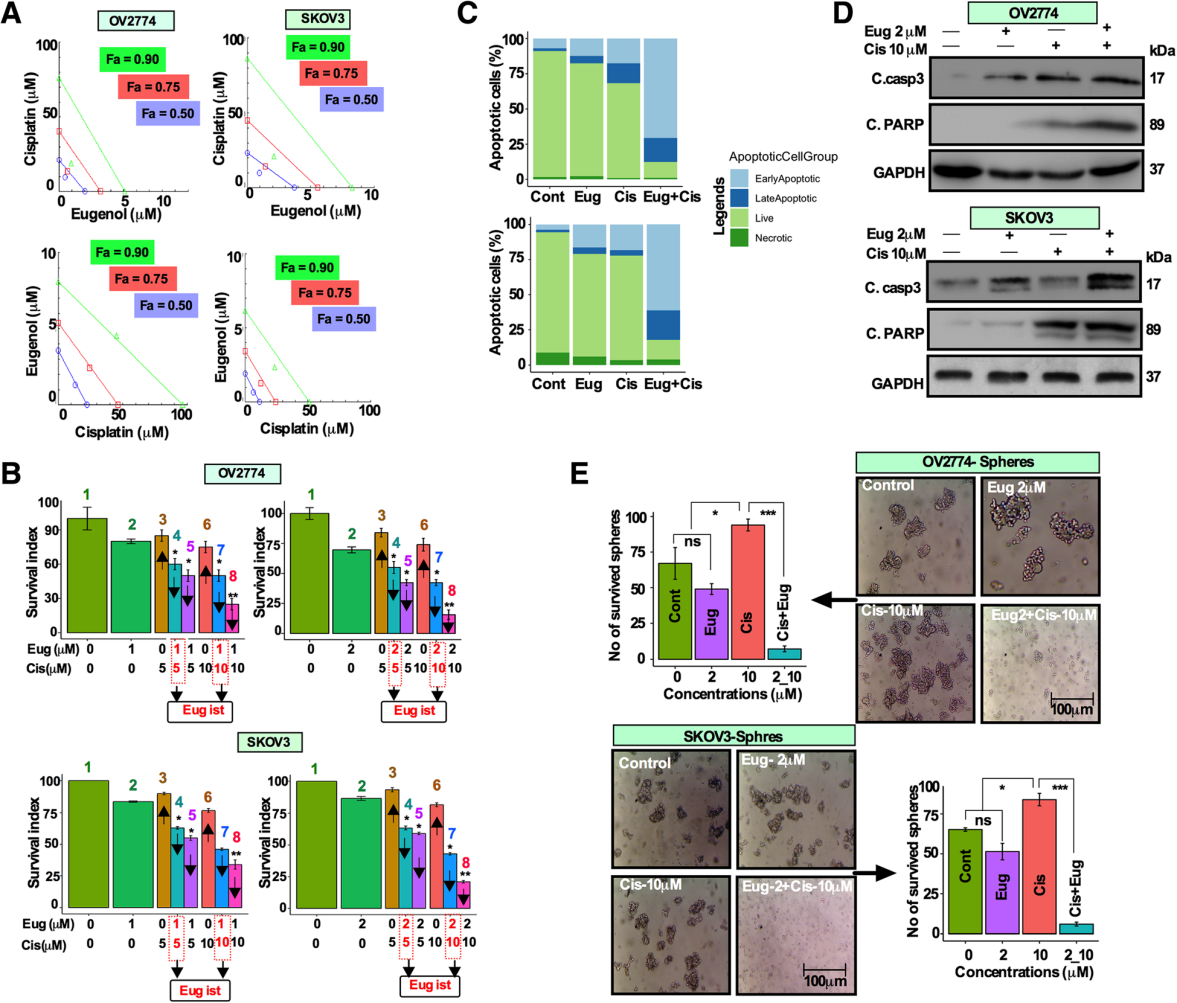
Eugenol sensitizes OC cells to cisplatin. **a** OV2774 and SKOV3 cells were treated with increasing concentrations of cisplatin and eugenol, for 72 h and dose response curves were determined by the WST-1 assay. Combination index (CI) and isobologram were generated using the CompuSyn software. The individual doses of cisplatin and eugenol to achieve 90% growth inhibition (green line, **Δ**-symbol, Fa = 0.90), 75% growth inhibition (red line, **□**-symbol, Fa-0.75) and 50% growth inhibition (blue line, **Ο**-symbol, Fa = 0.50) were plotted on the X and Y-planes. **b** Cells were treated as indicated, and cell survival was determined by the WST-1 assay. Significant differences were analyzed using Factorial ANOVA between cisplatin and eugenol single treatments. [Top and bottom left panel; Columns 4 and 7-eugenol at 1 μM constant, cisplatin 5 and 10 μM; top and bottom right panel; Columns 4 and 7 eugenol at 2 μM constant, cisplatin at 5 and 10 μM] (*n* = 3; mean +/− SD, * *p* 0.05, **p 0.01). **c** Exponentially growing cells were treated as indicated, and cell death was assessed by Annexin V/PI-associated flow cytometry. **d** Whole cell lysates were prepared from the indicated cells and were used for immunoblotting analysis using antibodies against the indicated proteins, while GAPDH was used as internal control. **e** Cells were treated with DMSO (control), cisplatin (10 μM), eugenol (2 μM), and the sequential combination of both drugs. Treated cells were cultured in ultra-low attachment plates for 10 days and spheres were photographed and the number of spheres were counted, analyzed and presented as bar graph (*n* = 3; mean +/− SD, *p 0.05, ***p 0.001)

First, we have tested the growth inhibitory effects of different concentrations of cisplatin and eugenol on two human ovarian cancer cell lines (OV2774; also known as MDAH-2774 and SKOV3) for different periods of time using the WST-1 assay. SKOV3 and OV2774 ovarian cell lines were extensively characterized previously [16, 17]. The growth inhibitory effect of cisplatin and eugenol alone were time- and concentration-dependent for both cell lines (Additional file 1: Figure S1A). The highest growth inhibition was observed by 72 h at 40 μM for cisplatin and 4 μM for eugenol (Additional file 1: Figure S1A). We then investigated the dose response of the combination of both drugs in two drug administration sequences, a) cisplatin (5, 10, 20, 30 and 40 μM) alone for 24 h followed by additional 48 h with eugenol (0.5, 1, 2, 3 and 4 μM) and, b) eugenol alone for 24 h followed by additional 48 h with cisplatin and cellular cytotoxicity and quantitative values of drug interaction combination index (CI) were determined using the method developed by Chou, 2006 [18]. In the sequence (a), the CI ranged from 0.971 to 0.081 for OV2774 cells and 0.956 to 0.183 for SKOV3 cells (Fig. 1a, Additional file 1: Figure S1B, Additional file 8: Tables S1A, S1B). In the sequence (b), the CI values for OV2774 cells was 0.834 for the combination doses of cisplatin 5 μM/eugenol 0.5 μM, and 1.192 for the combination doses cisplatin 20 μM/eugenol 2 μM. For SKOV3 cells, CI values ranged from 0.717 to 1.212 (Fig. 1a, Additional file 8: Table S1A, S1B). In the sequence (b), the CI values started to decline only at higher doses (cisplatin 30 μM)/eugenol 3 μM) and (cisplatin 40 μM/(eugenol 4 μM) (Fig. 1a, Additional file 1: Figure S1B, Additional file 8: Table S1B). These findings suggest that adding eugenol first at low concentrations generated antagonistic effects of the drugs, while adding cisplatin first followed by eugenol showed strong synergism.

To further test the effects of the combination, we have chosen two low-doses of cisplatin (5 and 10 μM), which killed between 20 and 30% cells (Additional file 1: Figure S1B). Therefore, when we combined cisplatin (5 and 10 μM) with a low dose of eugenol (1 μM), the proportion of cell death in OV2774 cells reached to 50 and 70% in a combination sequence (a) (Fig. 1b, top left panel; column 5 and 8 in bar graph). In contrast, when cells were treated with eugenol first followed by cisplatin (combination sequence b), cell death was 40 and 50% (Fig. 1b, top left panel; columns 4 and 7 in bar graph). Using similar combination sequence approach, we then tested if 2 μM of eugenol can further enhance the cell death with low-doses of cisplatin. In the sequence (b), when 2 μM of eugenol was added to 5 and 10 μM of cisplatin, cell death was enhanced to 51 and 62%, respectively (Fig. 1b, top right panel columns 4 and 7 in the bar graph). The percentage of cell death was further enhanced to 75 and 95% when cells were first treated with cisplatin followed by eugenol [combination sequence (a)] (Fig. 1b, top right panel; columns 5 and 8 in the bar graph). A similar response was seen with SKOV3 cells. Briefly, when cisplatin (5 and 10 μM) was combined with low-dose eugenol (1 μM), the proportion of cell death was 48 and 68% in a combination sequence (a) (Fig. 1b, bottom left panel, column 5 and 8 in the bar graph). However, in a combination sequence (b), the cell death was 35 and 52% respectively (Fig. 1b, bottom left panel, column 4 and 7 in the bar graph). The inhibition of cell death was more pronounced when 2 μM of eugenol was combined with 5 and 10 μM of cisplatin. The cell death was 35 and 48% (Fig. 1b, right panel, column 4 and 7 in the bar graph) and 55 and 90% respectively (Fig. 1b, bottom right panel, column 5 and 8 in the bar graph), when cells were first treated with cisplatin [combination sequence (a). Based on these results, we have chosen eugenol at 2 μM and cisplatin at 10 μM to test the pro-apoptotic effects of the combination using Annexin V/PI flow cytometry and immunoblotting. The synergistic effects were evident and the combination treatment increased the proportion of apoptotic cells to 87 and 82.5% in both OV2774 and SKOV3 cells (Fig. 1c). This was confirmed by showing clear increase in the level of cleaved-caspase 3 and cleaved PARP proteins, compared with single agents (Fig. 1d).

### Eugenol potently inhibits cisplatin-induced OCSCs

Cisplatin-induced CSC enrichment has been reported for OC cells [6]. Therefore, we assessed the effectiveness of the combination treatment on the sphere formation efficiency. While eugenol showed modest effect, cisplatin increased the number of spheres (Fig. 1e). Interestingly, the sphere formation efficiency was abolished by the combination treatment (Fig. 1e). This suggests that eugenol can suppress the cisplatin-related enrichment of OCSCs.

### Hes1 ectopic expression promotes CSC characteristics and resistance to cisplatin

**Fig. 2:**
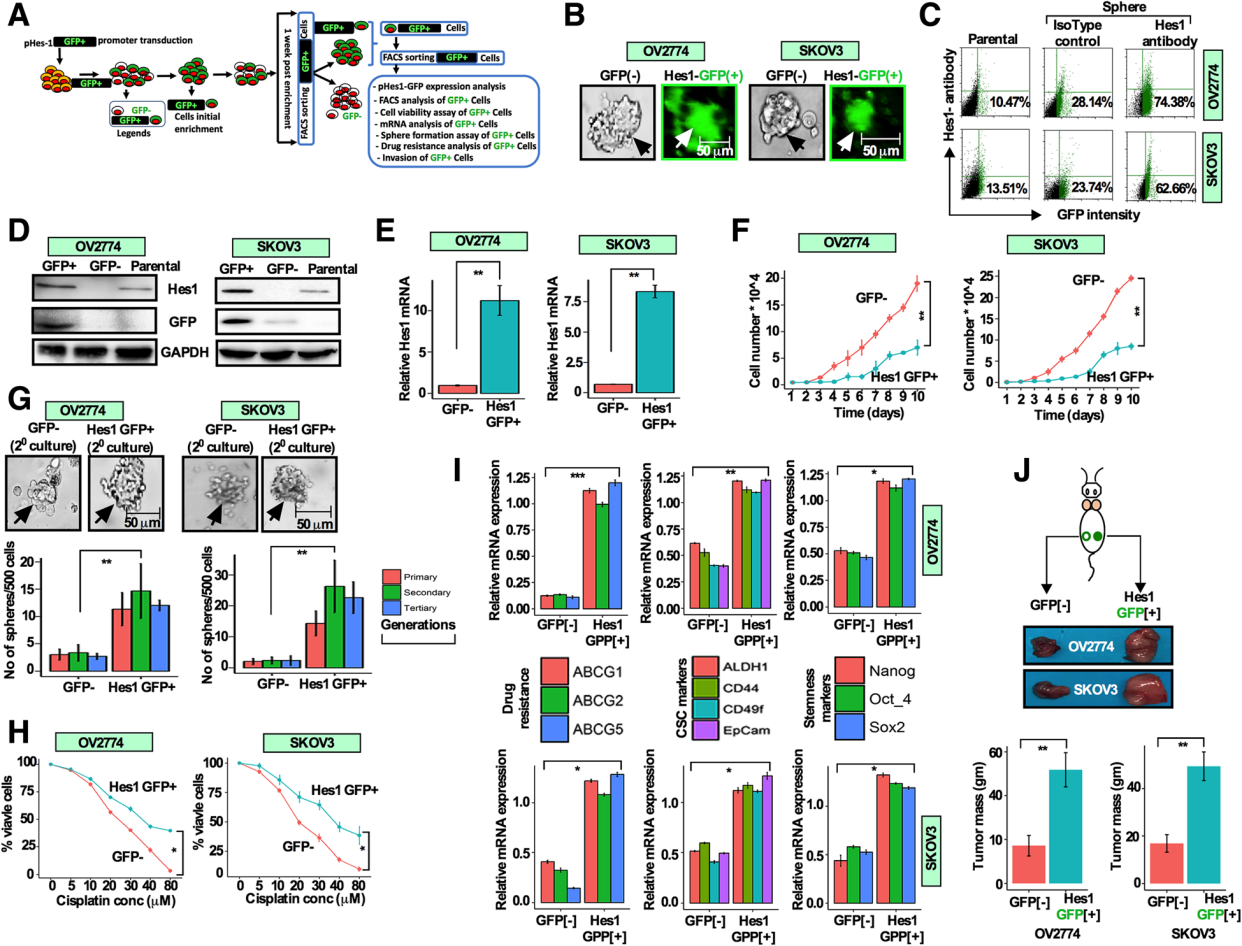
Hes1 ectopic expression promotes CSC characteristics and resistance to cisplatin. **a** Schematic illustration describing the transduction procedures of OV2774 and SKOV3 cells with the pHes1dEGFP reporter and data analysis. **b** Transduction of cells with the pHes1d2EGFP reporter and representative photomicrographs of parental cells and spheres. **c** Parental and Hes1GFP(+) cells were cultured, dissociated from spheres, labelled with anti-Hes1 antibody and isotype control and analyzed by flow cytometry. **d** Cell lysates were prepared from the indicated cells and analyzed by immunoblotting. **e** Total mRNA was purified and the level of the *HES1* mRNA was assessed by qRT-PCR, (*n* = 3, mean +/− SD; Students *t*-Test; ** *p* 0.01). **f** Cell growth of the indicated cells was analyzed by the WST1 assay. (n = 3, mean +/− SD; Students t-Test; ** *p* 0.01). **g** Sphere formation ability of Hes1GFP(+) and GFP(−) cells was analyzed at serial generations. Photographs are from secondary cultures. Sphere numbers were counted and presented as bar graph (n = 3, mean +/− SD; Students *t*-Test; ** *p* 0.01). **h** Cells were treated with increasing concentrations of cisplatin and cytotoxicity was assessed by the WST1 assay. (n = 3, mean +/− SD; Students *t*-Test; * *p* 0.05). **i** Total RNA was purified from Hes1-GFP-positive and GFP-negative cells, and then the expression of the indicated genes was assessed by qRT-PCR (n = 3, mean +/− SD; Students *t*-Test;*p 0.05, ** *p* 0.01). **j** Transfected cells were FACS-sorted as described in **(a**; **b)**. Sorted GFP(+/−) cells were transplanted subcutaneously in mice (*n* = 5/group). Resected tumors were photographed 8 weeks after implantation. Bar graph showing the tumor weight in each group (Students *t-*Test; mean +/− SD **p 0.01)

Studies have shown that Hes1, a downstream effector of the Notch signaling, is closely involved in cancer progression, chemoresistance, promotes self-renewal and tumor initiation in various cancers [19]. However, the role of Hes1 in OCSCs remains mostly unclear. Therefore, to evaluate the importance of the Notch-Hes1 pathway in OC, we first assessed the genetic alterations of Hes1, Hey1, Notch1, Notch2 and Notch3 from cBioportal for Cancer Genomics data portal. Hes1 locus is amplified in 2–25% in breast and 2–22% in ovarian cancer, followed by Hey1, Notch2 and Notch3 (Additional file 2: Figures S2A-S2E). To get more insight into the role of Hes1 in regulating OCSC functions and response to cisplatin, we have ectopically expressed human Hes1 in a promoter driven green fluorescence protein (GFP) reporter system pHes1d2EGFP [15] in OV2774 and SKOV3 cells (Fig. 2a). After transduction, Hes1GFP^**+**^ cells were sorted and then were cultured in CSC medium for 10 days (Fig. 2b). Spheres were enzymatically isolated and analyzed by flow cytometry to isolate Hes1GFP(+) and GFP(−) cells and identified approximately 74 and 63% of Hes1GFP^**+**^ cells in OV2774 and SKOV3 cells (Fig. 2c), however, the parental cells contained only 10 and 13% of Hes1^+^ cells, respectively (Fig. 2c). However. The expression of the Hes1 protein and mRNA in Hes1GFP^**+**^ and GFP^**−**^ cells were confirmed by Western blotting and qRT-PCR (Figs. 2d; e). The Hes1GFP^**+**^ cells grew slower in complete medium (medium supplemented with 10% FBS) (Fig. 2f) and showed higher sphere formation ability than GFP^**−**^ cells in supplemented CSC medium (Fig. 2g). This prompted us to assume that Hes1GFP^**+**^ cells are likely to be resistant to cisplatin than GFP^**−**^ cells. To test this, Hes1GFP^**+**^ and GFP^**−**^ cells were treated with increasing concentrations of cisplatin. Hes1GFP^**+**^ cells showed higher cell viability than GFP^**−**^ cells (Fig. 2h). Furthermore, the proportion of cleaved caspase-3 positive cells was higher in GFP^**−**^ cells relative to Hes1GFP^**+**^ cells when treated with cisplatin (10 μM) (Additional file 2: Figure S2F). Moreover, multi-drug resistance markers ABCG/MDR1, ABCG2 and ABCG5; CSC markers CD44, ALDH, EpCam and CD49f, and pluripotency markers Nanog, Oct3/4 and Sox2 were all highly expressed in Hes1GFP^**+**^ cells as compared to GFP^**−**^ cells (Fig. 2i). Additionally, Hes1GFP^**+**^ cells were more invasive than the parental and GFP^**−**^ cells (Additional file 2: Figure S2G). To confirm the in vitro results in animal model, Hes1GFP^**+**^ cells were isolated as described in Fig. 2a and implanted subcutaneously into nude mice (*n* = 5/group) and revealed the greater ability to form tumors by Hes1GFP^**+**^ compared to the GFP^**−**^ cells (Fig. 2j). These results indicate that increase in the expression of Hes1^**+**^ cells promote CSC traits, and resistance to cisplatin of OC cells.

### Combination treatment prevents self-renewal, CSC enrichment and invasive behaviour in OC cells

To investigate the effects of combination treatment on OCSCs, we have first analyzed the CD44 expression and ALDH activity in Hes1-GFP^+^ and GFP^−^ sorted OV2774 and SKOV3 cells. Hes1-GFP^+^ cells were grown in supplemented CSC medium to enrich the CSC activities and sorted as described in Fig. 2a. Immunoblot analysis from sorted Hes1-GFP^+^ and GFP^−^ populations showed that CD44 and ALDH1 expressions were distinguishable in both cell lines (Additional file 3: Figure S3A). We then analyzed the self-renewal capacity of sorted CD44^+^ and CD44^−^ cells. It revealed that CD44^+^ cells are more self-renewal capable than CD44^−^ cells (Additional file 3: Figure S3B). Furthermore, ALDH^+^ cells are more self-renewable capable than ALDH^−^ counterparts (Additional file 3: Figure S3C). In addition, the size and the number of spheres were significantly reduced in CD44^−^/ALDH^−^ fraction as compared to their CD44^+^/ALDH^+^ populations (Additional file 3: Figure S3D). When CD44^+^ and ALDH^+^ cells were both removed simultaneously by FACS, cells lost the sphere formation capacity (Additional file 3: Figure S3E, S3F). These results suggest that CD44^+^/ALDH^+^ subpopulations were the most efficient in sphere formation among Hes1-GFP^**+**^ cells.

**Fig. 3:**
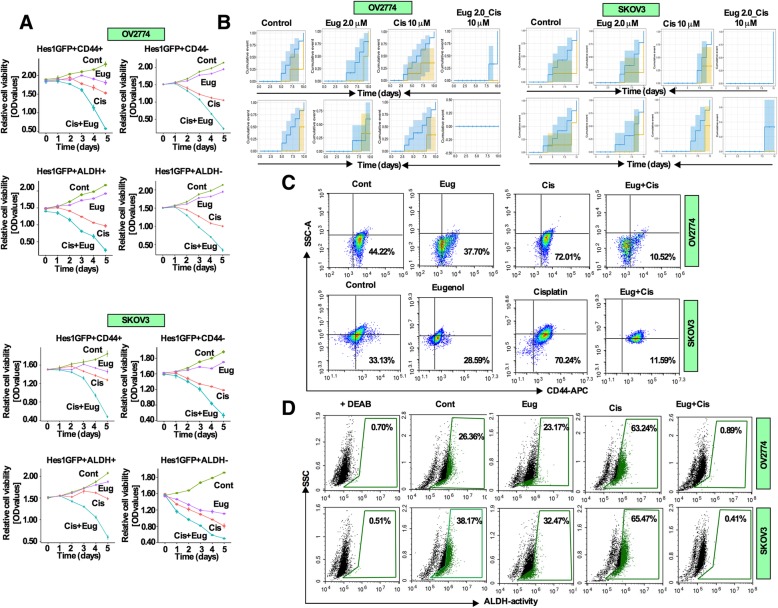
Combination treatment reduces the proportion of CD44^+^ and ALDH^+^ OCSC cells. **a** Sorted Hes1GFP^**+**^-CD44^**+/−**^ /Hes1GFP^**+**^-ALDH^**+/−**^ cells were cultured in CSC medium (96-well plate), and were treated with cisplatin (10 μM), eugenol (2 μM) and the combination of both drugs for 5-days. The viability of spheres was determined by the WST-1 assay. **b** Sorted Hes1GFP^**+**^CD44^**+**^ and Hes1GFP^**+**^ALDH^**+**^ cells were treated with vehicle (control), cisplatin, eugenol and combination of both agents for 72 h, trypsinized and cultured as spheres (5000 cells/well) in CSC medium for 10 days. Sphere forming ability was monitored routinely under the microscope and counted and recorded the number of spheres from each group. Data are presented as “cumulative event” and presented as survival stair graph. **c** Cells were treated with DMSO (control), cisplatin (10 μM; 72 h), eugenol (2 μM; 72 h) or the combination of both drugs (cisplatin 24 h, followed by eugenol for additional 48 h; total 72 h). CD44-FITC antibody was used to sort cells using the NovoCyte flow cytometer. **d** The ALDEFLOUR assay was performed to identify the CSC populations following treatment as in (**c**). ALDH specific inhibitor DEAB was used as negative control, ALDH^**+**^ cells are shown in cells residing in framed area analyzed using NovoExpress software

Next, we hypothesized that combination treatment could specifically target CD44^**+**^/ALDH^**+**^ populations. To address this, sorted CD44^+/−^ and ALDH^+/−^ cells were treated with DMSO (control), cisplatin and eugenol either alone or in combination for 5 days, and then cell viability was assessed by the WST-1 assay. The combination treatment significantly inhibited the growth of CD44^+^/ALDH^+^ cells compared to single agents (Fig. 3a). Next, we assessed the effectiveness of combination treatment on self-renewal ability in CD44^+^/ALDH^+^ fractions. Cells were sorted, cultured (adherent culture) and treated for 72 h, trypsinized and cultured in the supplemented CSC medium for 10 days (sphere culture). While both control and eugenol treated cells were able to form spheres overtime (blue line: CD44^+^**/**ALDH^+^ yellow line: CD44^−^**/**ALDH^−^), treatment with cisplatin increased the number of spheres within the same time period (Fig. 3b). In contrast, the combination treatment started to reduce the ability of sphere formation as early as 3–4 days in both cell populations (Fig. 3b). We then assessed the effects of combination treatment in the changes of CD44 and ALDH fractions by flow cytometry. The combination treatment significantly reduced the fractions of CD44^+^ and ALDH^+^ subpopulations (Fig. 3c; d). While eugenol eliminated CD44^+^ population by only 6% in OV2774 cells (statistically not significant), the proportion was increased by cisplatin to 72.50%. However, the combination treatment strongly reduced the proportion of CD44^+^ cells to 10.5% (Fig. 3c). Likewise, cisplatin increased the proportion of CD44^**+**^ cells to 73.18% in SKOV3 cells, while co-treatment reduced this proportion to 11.5% (Fig. 3c). Similarly, the ALDH activity increased by cisplatin from 22.58% (control) to 40.70% in OV2774 cells and 15.75 to 41.99% in SKOV3 cells (Fig. 3d). In contrast, co-treatment significantly reduced ALDH activity to 4.23% in OV2774 cells and 3.33% in SKOV3 cells (Fig. 3d). These results suggest that combination treatment effectively reduced the CD44^+^ cells and ALDH activity in OCSC populations.

### Combination treatment reduces the resistant side populations, and inhibits the expression of drug resistance, CSCs and pluripotency-related genes

**Fig. 4:**
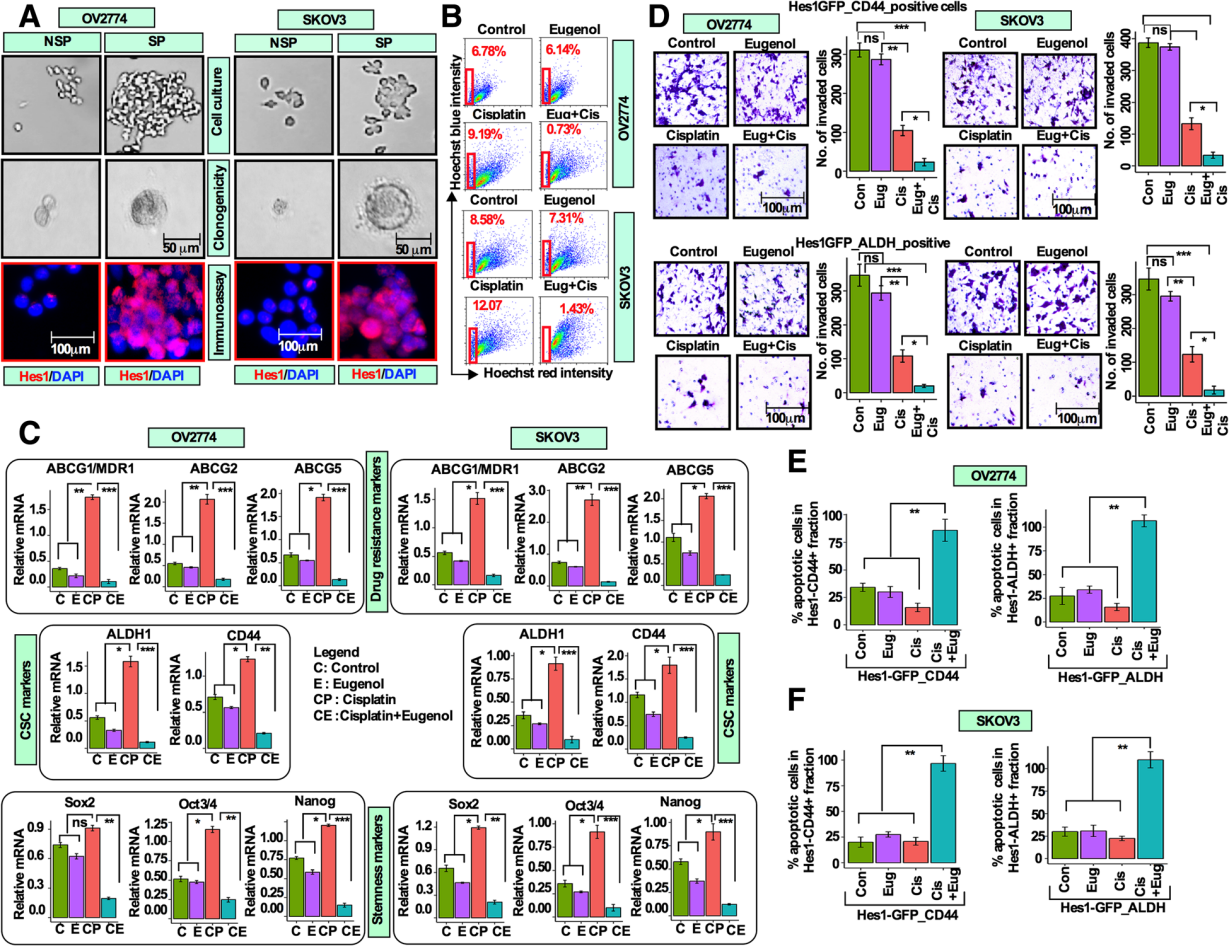
Combination treatment supresses cisplatin-induced side populations and inhibits the expression of drug resistance, CSC, and pluripotency-related genes. **a** [top panel] Cells were labelled with Hoechst 33342 dye and separated by FACS and sorted 10,000 SP and NSP cells were cultured in 24-well plates and after 5-days SPs and NSPs were photographed. [middle panel] Sorted SP and NSP cells were cultured in agarose medium and cells were allowed to grow for 3 weeks and colonies were photographed. [bottom panel] SP and NSP cells were stained for Hes1 by immunofluorescence (red: Hes1, blue: DAPI). **b** Cultured cells were treated with DMSO (control), eugenol (2 μM), cisplatin (10 μM), and combination of both for 72 h, and then labeled as described in **(a)** and SP cells were analyzed by flow cytometry. The numbers in the boxes indicate the proportion of SP cells. **c** Cells were treated as **(b)** and the expression of the indicated genes was assessed by qRT-PCR (*n* = 3, mean +/− SD; ns = not significant; *p 0.05; **p 0.01; ***p 0.001). **d** CD44^+^/ALDH^+^ cells were treated as **(b)** and transwell invasion assay was performed for 24 h. Invaded cells were stained with 10% crystal violet dye. Left panels, light microscope photographs (40x magnification). Right panels, bar graphs showing numbers of invaded cells. (n = 3, mean +/− SD; ns = not significant; *p 0.05; **p 0.01; ***p 0.001). **e** and **f** Cells were treated as **(b)**, and then were stained with Annexin-V and propidium iodide. Cell death was assessed by flow cytometry, and the proportions of apoptotic cells were presented as bar graphs. (n = 3; mean +/− SD; **, *P* 0.01)

Several studies have shown that side populations (SPs) exhibit CSC-like properties and are responsible for drug resistance and recurrence [20]. SPs are able to efflux DNA binding dye Hoechst 33342 out of the cell membrane and enriched with CSC populations in OC [21, 22]. We therefore sought to enrich and purify SPs from OV2774 and SKOV3 cells by FACS after verapamil treatment (verapamil is an inhibitor of several verapamil-sensitive ABC-transporters). In the SKOV3 cells, the proportion of SPs in the control cells was 8.17%, whereas this proportion was reduced to 1.07% when cells were treated with verapamil (Additional file 4: Figure S4A). When characterizing sorted SPs and non-SPs (NSPs), SPs were able to divide indefinitely, and formed cobblestone epithelial like colonies, highly tumorigenic when grown in agarose medium and positive for Hes1 (Fig. 4a). On the other hand, NSPs ceased proliferating within 3 weeks and were less tumorigenic and mostly negative for Hes1 (Fig. 4a). To analyze combination effect on these SPs, cells were treated with either cisplatin and/or eugenol alone or in combination, and then SP fractions were analyzed by FACS. The proportions of SPs were 6.78 and 8.58% for OV2774 and SKOV3 cells (Fig. 4b). While SPs were significantly unaffected by eugenol (6.14% for OV2774 and 7.31% for SKOV3), cisplatin treatment increased the proportion of SPs to 9.19% in OV2774, and 12.07% in SKOV3 cells (Fig. 4b). Interestingly, these inductions were reduced by cotreatment to 0.73% in OV2774 and 1.43% in SKOV3 cells. Next, we investigated the combination effect on the expression of several drug resistance, CSCs and pluripotency markers by qRT-PCR. While the effect of eugenol was minimal on ABCG1/MDR1, ABCG2 and ABCG5, these genes were significantly up-regulated when exposed to cisplatin. In contrast, these genes were sharply down-regulated by cotreatment (Fig. 4c). Similar results were obtained for the CSCs and the pluripotency cohort (Fig. 4c). Identical results were observed for all these genes in SKOV3 cells (Fig. 4c). These findings indicate that, SPs that are capable of self-renewal and drug resistance could effectively be targeted by the combination treatment. Furthermore, combination treatment significantly inhibited the invasion/migration abilities of both CD44^+^ and ALDH^+^ sub-populations compared to single agents (Fig. 4d, Additional file 4: Figure S4B). We then assessed the apoptotic response in the sorted CD44^+^ and ALDH^+^ cells and found that while single agents had only partial effects on these cells, the combination treatment significantly increased the proportion of apoptotic cells to 90% in both CD44^+^ and ALDH^+^ cells (Fig. 4e).

We then analyzed the correlation between drug resistance, CSC and pluripotency cohorts with Hes1 using results obtained from qRT-PCR and analyzed using “R” with CORRPLOT package. In most instances, Hes1 is correlated with all markers analyzed and “r” values ranging from 0.94 (Sox2 vs ABCG2) to 0.99 (ABCG2 vs Nanog) and so on. For example, Hes1 was highly positively correlated with ABCG2 (r = 1.0, *p* 0.001) and Nanog (r = 0.98, *p* 0.002) in OV2774 and SKOV3 cells (Additional file 4: Figure S4C, related Additional file 8: Table S4C-i-iv).

### Cisplatin and eugenol cotreatment targets OCSCs by inhibiting the Notch pathway

**Fig. 5:**
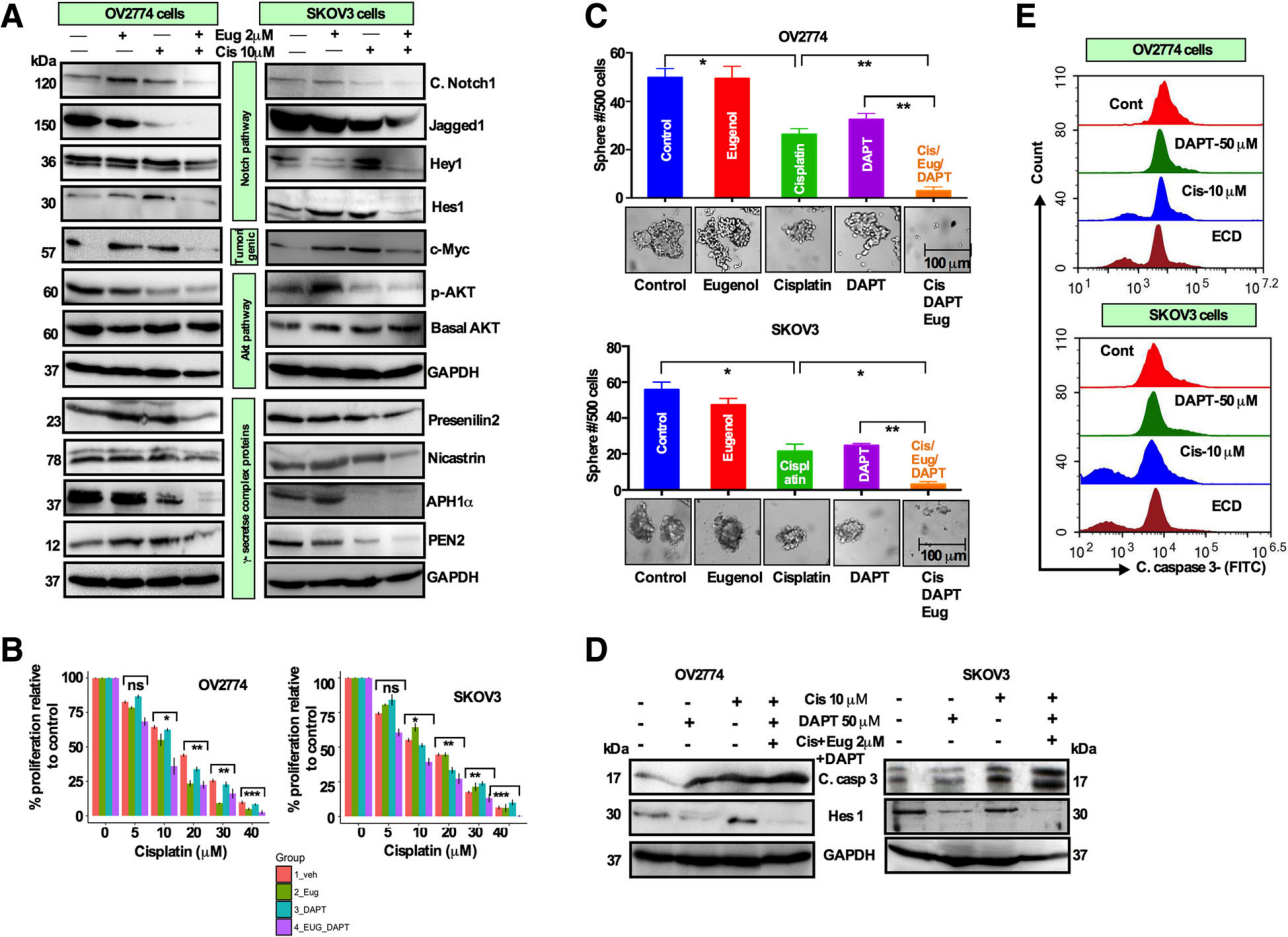
Cisplatin and eugenol combination treatment inhibits the Notch signaling pathway. **a** Whole cell lysates were prepared from sorted spheres, and then were treated with DMSO (control), cisplatin (10 μM), eugenol (2 μM) and combination of both, and incubated for 72 h, and immunoblotting analysis was performed using antibodies against the indicated proteins. **b** Cells were treated with increasing concentrations of cisplatin for 24 h followed by eugenol (0.5, 1, 2, 3 and 4 μM) and DAPT (10, 20, 30, 40, 50 and 100 μM) and cell proliferation was analyzed by the WST-1 assay (n = 3, mean +/− SD; *p 0.05, **p 0.01, ***p 0.001). **c** Growth of spheres were determined after treatment of spheres with cisplatin (10 μM), DAPT (50 μM) and combination of cisplatin/eugenol (2 μM)/DAPT and sphere numbers were analyzed and presented as bar graphs (n = 3, mean +/− SD; * *p* 0.05, **p 0.01). **d** Cells were treated as indicated and whole cell lysates were used for immunoblotting analysis utilizing antibodies against the indicated proteins. **e** Cells were treated as **(c)** and the proportion of cells expressing cleaved caspase-3 was determined by flow cytometry

To explore the Notch-dependent cisplatin mediated drug resistance and CSC regulation, we investigated the possible implication of the Notch pathway in the cisplatin and eugenol treatment-dependent targeting of OCSCs. To explore this, sorted Hes1^**+**^ cells were allowed to grow in the CSC medium, and then were treated with cisplatin and eugenol either alone or in combination. Spheres were collected and the expression of the Notch pathway proteins was analyzed by immunostaining. The expression intensity of Hes1, CD44 and ALDH was significantly reduced in the combination-treated cells. While eugenol alone did not alter the expression intensity, cisplatin alone increased the expression intensity in both OV2774 and SKOV3 cells (Additional file 5: Figure S5A and S5B). In line with these results, the immunoblot results showed the levels of Hey1, Hes1, cleaved Notch1 and Jagged1 were all reduced by cotreatment as compared to single treatments (Fig. 5b). c-Myc is a direct downstream target of Notch1 and preferentially induces the Akt signaling [23, 24]. c-Myc and the phospho-Akt were downregulated by cotreatment without affecting the basal level of AKT (Fig. 5b). Additionally, we determined whether the combination affected the γ-secretase complex consisting of Prenisillin-2, Nicastrin, APH1alpha and PEN2. All γ-secretase complex proteins were downregulated by the cotreatment compared to single agents (Fig. 5b). Furthermore, adding DAPT, a γ-secretase complex inhibitor, further inhibited cell proliferation (Fig. 5c), self-renewal and sphere formation capacity (Fig. 5d), while induced apoptosis (as evidenced by Western blot and flow cytometry) and reduced Hes1 expression (Fig. 5e, f). Together, these results indicate that the combination treatment targets OCSCs by inducing apoptotic activity as well as through inhibition of the Notch signalling pathway.

### In vivo cotreatment represses tumor growth and increases tumor-free survival

**Fig. 6:**
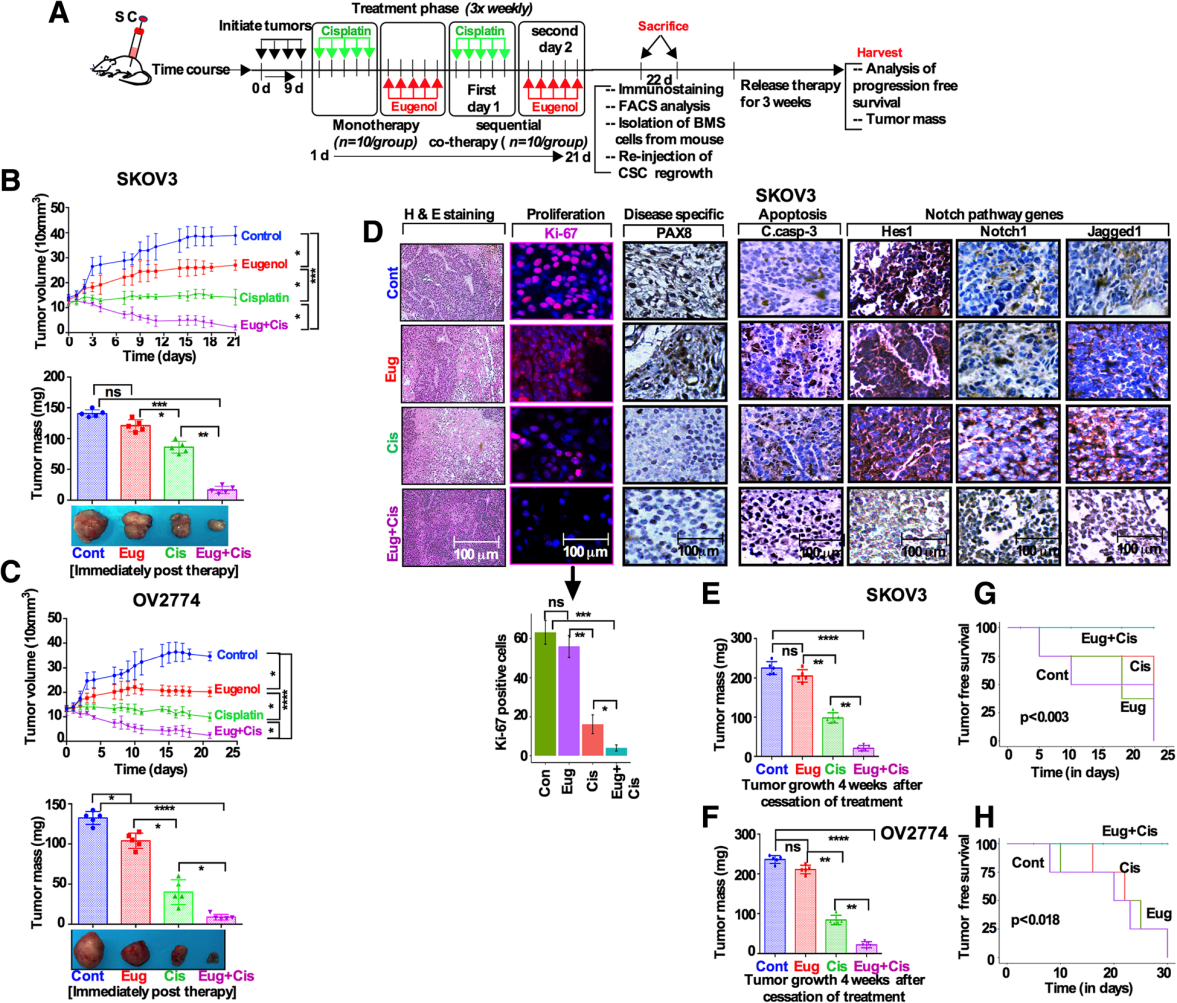
Eugenol sensitizes ovarian cancer cells to cisplatin in vivo. **a** Experimental design: Mice (*n* = 10/group) harboring tumors were treated with DMSO (control), cisplatin, eugenol and both drugs for 3 weeks. Xenografts were harvested from half the mice at the completion of treatment (*n* = 5/group), and the remaining xenografts (n = 5/group) were harvested 1 month later. **b** and **c [**Top panels] During the treatment period tumor volumes were measured **[**Bottom panels] weighted tumor masses at harvest. ‘*p*’ values from One-way ANOVA on graphs and Eug vs Eug + Cis = *p 0.05; ***p 0.001 and Cis vs Eug + Cis = ****p 0.0001, respectively by unpaired two-sided t-test. **d** H&E as well as immunostaining using antibodies against the indicated proteins. The graph bar represents quantification of the Ki-67 immunofluorescence. (n = 3, mean +/− SD; Students *t*-Test;*p 0.05, ** *p* 0.01, *** *p* 0.001). **e** and **f.** Tumor masses at harvest. ‘*p*’ values from Eug vs Eug + Cis = **p 0.01; and Cis vs Eug + Cis = **p 0.01, respectively by unpaired two-sided t-test. **g** and **h.** Tumor-free survivals were determined following 30-days post cessation of treatment (Log-rank test *p* values: 0.003 and 0.18)

To evaluate the efficacy of combination treatment on tumor growth and OCSCs in vivo, we utilized xenograft models. Mice carried xenografts from SKOV3 and OV2774 cells injected as single inoculums in the dorsal site of each mouse (*n* = 10/group; Fig. 6a). Inception of tumors was first confirmed, and then animals were randomized and treated with DMSO (control), eugenol, cisplatin or both for 3 weeks in a drug administration sequence described in Fig. 6a. Xenografts were monitored for treatment efficacy at the completion of treatment (*n* = 5/group) and disease progression (n = 5/group) 4 weeks after cessation of treatment. Tumor volume and weight were regressed in eugenol and cisplatin treatment group, but significant regression was observed in the cotreatment group (Fig. 6b; c). This indicates that eugenol strongly potentiates tumor growth inhibitory effect of cisplatin. Tumor sections were stained with H&E and were also immunostained for proliferation, disease specific and the Notch pathway markers. The cotreatment strongly reduced the expression of Ki-67 and PAX8 relative to the control and monotherapy groups (Fig. 6d; Additional file 6: Figure S6A). Similar effect was also observed on Hes1, Notch1 and Jagged1 (Fig. 6d; Additional file 6: Figure S6A). Furthermore, cleaved caspase-3 level was induced in cotreatment group as compared to controls (Fig. 6d; Additional file 6: Figure S6A). Progression-free survival is an important tool for evaluating the efficacy of a treatment and response to therapy. We therefore analyzed the tumor mass and the progression of the tumors 4 weeks after cessation of treatments (Fig. 6a). The treatment response was durable without gaining tumor mass in the co-therapy group 4 weeks post-treatment (Fig. 6e, f). In the cotreatment group, tumors were barely detectable in all mice, and only small foci of tumor was detected in one mouse (Fig. 6e, f). Tumor progression was observed in all monotherapy treated mice within 10 and 15 days after cessation of treatment except for the cotreatment group (Fig. 6g, h).

### Cisplatin/eugenol combination treatment strongly suppresses OCSC self-renewal and ameliorates disease-free survival of animals

**Fig. 7:**
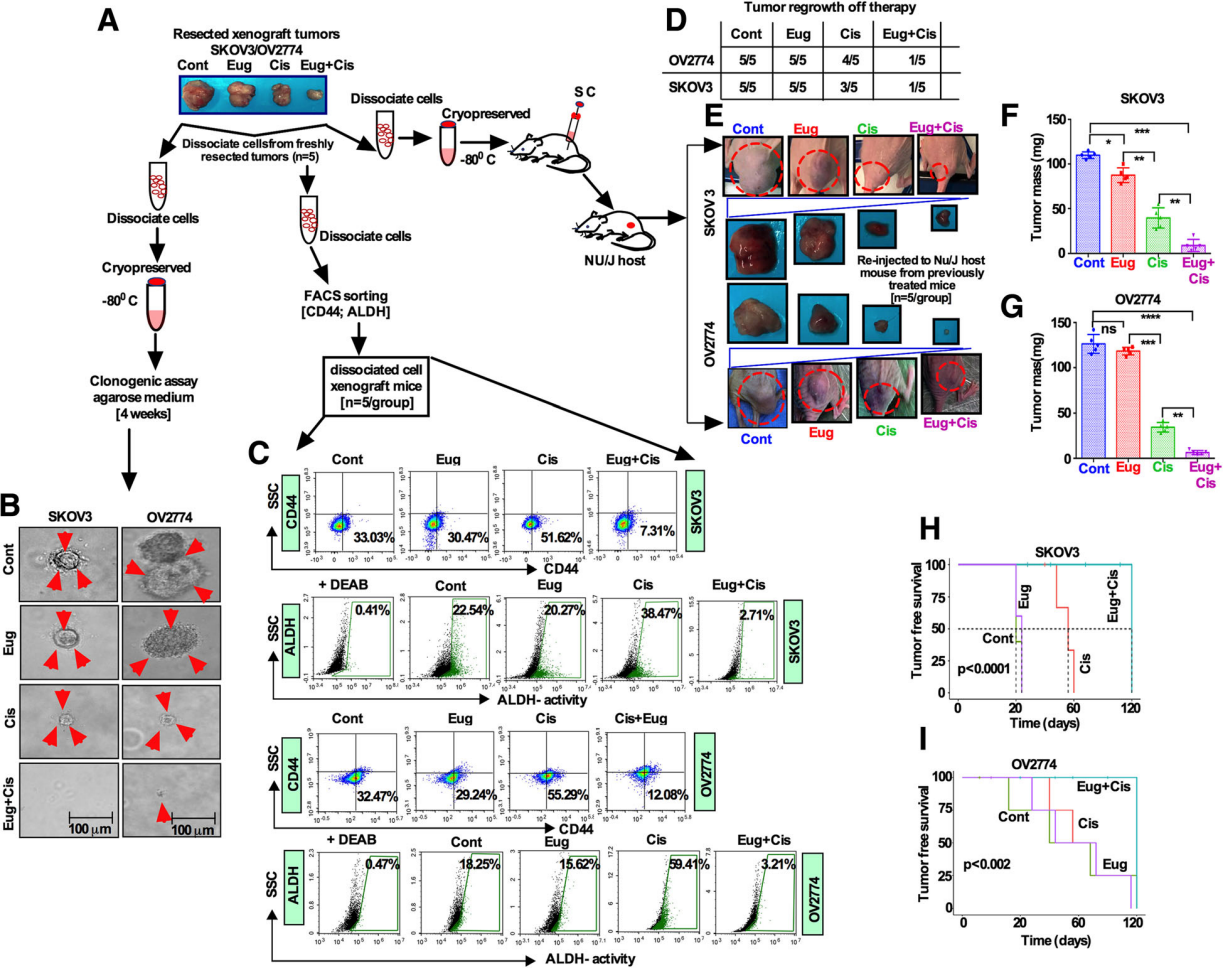
Eugenol/cisplatin combination strongly suppresses OCSC self-renewal and ameliorates disease-free survival of animals. **a** Experimental design: Freshly dissociated live-banked cells from tissues harvested from control, eugenol-, cisplatin- and combination-treated mice as described in Fig. 6 were either left unsorted or were sorted before further analysis as shown. **b** Dissociated cells were cryopreserved, and then were cultured for 3 weeks in a semi-solid agarose medium for colony formation analysis. **c** Dissociated cells from freshly harvested xenograft tumors were FACS sorted for CD44 and ALDH. **d** and **e.** Freshly dissociated cells from xenograft tumors were re-implanted into Nu/J mice (n = 5) for 8 weeks as described in Fig. 6a and the numbers of palpable tumors are shown in Table **(d),** and **e** Representatives of harvested xenograft tumors from control and treatment groups. **f** and **g.** Tumor masses [at harvest, ‘*p*’ values from Eug vs Eug + Cis = **p 0.01; and Cis vs Eug + Cis = ***p 0.001, respectively by unpaired two-sided *t*-Test]. **h** and **i**. Tumor-free survival analysis (Log-rank) of OV2774 (*p* = 0.0001) and SKOV3 (*p* = 0.002) xenografts

To assess the long-term effects of the cotreatment and the self-renewal capacity of OCSCs, equal number of dissociated unsorted cells from excised tumor xenografts were cultured for 3 weeks in a semi-solid agarose medium. While cells from control and eugenol treated xenografts grew robust colonies, cells from cisplatin-treated tumors had slower but steady growth and grew small colonies. On the other hand, no colonies were formed from tumors treated with combination (Fig. 7a, b). This indicates that cotreatment abolished the self-renewal capacity of OCSCs. Although, dissociated tumor cells from co-treated SKOV3 xenografts significantly reduced the proportion of CD44 population (4.97%) and ALDH (2.05%) activity in these tumors, these proportions remained higher in the controls and monotherapy treated tumors (Fig. 7c). Identical results were obtained for the tumors from OV2774 cells (Fig. 7c). To confirm these results in vivo, the dissociated cells from previously treated mice (refer to Fig. 6a) were re-implanted into mice subcutaneously (*n* = 5/group) and left for 16 weeks without treatment. While, tumor cells from untreated and monotherapy-treated mice regrew and progressed, only small foci of tumor (1 out of 5 mice) were detected in tumor cells from co-therapy treated mice group (Fig. 7d, e, f, g). In contrast, animals inoculated with tumor cells derived from cotherapy-treated animals showed significantly better tumor-free survival as compared to the control group (Fig. 7h, i).

## Discussion

Although cisplatin and its derivatives have been widely used for OC management, their therapeutic success was limited due to acquired resistance and disease relapse for the majority of OC patients [5, 25]. Recently, it has become clear that this resistance to platinum-based chemotherapies is mainly due to OCSC enrichment [26, 27]. In the present study, we have first shown that eugenol, a natural phenolic compound, has cytotoxic and pro-apoptotic effects against OC cells both in vitro and in vivo. Moreover, when added to cisplatin-pre-treated cells, in a sequential combination, eugenol synergistically enhanced the anti-proliferative, cytotoxic and pro-apoptotic effects of cisplatin in vitro and in tumor xenografts. Furthermore, these co-treated OC cells were significantly less invasive and migratory than those treated with single agents. These results indicate that eugenol can potentiate the anti-OC effects of cisplatin. Likewise, we have recently shown similar synergism between cisplatin and eugenol on triple negative breast cancer cells [12]. However, in this case the combination was rather simultaneous, while for OC cells the best results were obtained with sequential combination: cisplatin followed with eugenol. This indicates that cisplatin and eugenol may synergistically act on 2 different pathways or mechanisms. Indeed, while cisplatin effects are mainly mediated through DNA damage, eugenol is a potent inhibitor of cell cycle and promoter of apoptosis through strong E2F1 down-regulation [28]. Like eugenol, other phytomolecules such as withaferin, berberine and genistein also sensitized OC cells to cisplatin [29–31]. This indicates that potentiation of the cytotoxic effects of cisplatin against OC cells is possible by natural molecules, which can enhance the efficiency and reduce the side effects of this potent anti-cancer molecule.

More importantly, eugenol suppressed cisplatin-dependent promotion of stemness in OC cells and the consequent enrichment of OCSCs. Indeed, while cisplatin increased tumorsphere formation and survival capacities in OC cells, addition of eugenol to cisplatin significantly reduced these capacities through targeting CD44^high^ and ALDH^high^ subpopulations. To confirm this, we have also shown that the combination treatment efficiently targets side population cells, which are known to be highly resistant to cisplatin and an enriched source of OCSCs [20]. These effects were further confirmed in vivo on tumor xenografts by showing strong inhibitory effect of the combination therapy on self-renewal capacity of tumor initiating cells. This indicates that cisplatin/eugenol-dependent targeting of OCSCs could be of great therapeutic value. Indeed, we have shown here, in a preclinical study that the cotherapy inhibited tumor growth, promoted apoptosis, reduced tumor volume and ameliorated disease-free survival of animals as compared to monotherapies. Additionally, while cells from the residual tumors harvested from animals treated with single agents conserved their self-renewal and tumor initiation capacities, those harvested from animals treated with the combination have lost most of these abilities. This has clearly shown the great value of this combination in targeting OCSCs in vivo as well, which indicates its power to be used for the treatment of OC patients. Likewise, Ma et al. [32] have recently shown that the combination of thioxodihydroquinazolinone with cisplatin can eliminate OCSC-like cells. This indicates that the pro-OCSCs effects of cisplatin can be suppressed by various molecules.

At the molecular level, drug resistance is also due to the high expression of drug efflux pumps, which reduce the intracellular effects of the drugs. While the effect of eugenol was only marginal on the ABC transporters ABCG1/MDR1, ABCG2 and ABCG5, these genes were significantly up-regulated when exposed to cisplatin. Importantly, eugenol strongly suppressed this cisplatin-dependent upregulation of these genes, and significantly reduced their levels compared to controls. This shows that combining cisplatin with eugenol could constitute an effective strategy to reverse drug resistance through preventing drug efflux.

It has been previously shown that cisplatin-dependent enrichment of the OCSC population is mediated through increase in the expression of DNA polymesase eta, the upregulation of c-Myc and EZH2 or the activation of the Notch pathway. In fact, inhibition of each one of these pathways sensitized OC cells to cisplatin and suppressed cisplatin-related promotion of stemness in OC cells [10, 33, 34]. In the present study, we have also shown that the activation of the Notch pathway through Hes1 upregulation promotes stemness in OC cells and enhances their resistance to cisplatin. Furthermore, we present clear evidence that the cisplatin/eugenol combination is a potent inhibitor of the Notch pathway and its consequent promotion of OCSCs both in vitro and in tumor xenografts. While the role of Hes1 in stemness, metastasis and drug resistance is well established for various types of tumors, this is the first demonstration that Hes1 is also implicated in OCSCs and their resistance to cisplatin. Furthermore, McAuliffe et al. have shown that targeting the Notch pathway through γ-secretase specific inhibition depleted OCSCs and sensitized OC cells to platinum via enhancing the cellular response to DNA damage [10]. Together, these results indicate that inhibition of the Notch signaling pathway could constitute a highly effective strategy to target OCSCs and overcome resistance to platinum-based treatments in the clinical setting.

## Conclusions

The present findings present the sequential combination of cisplatin and eugenol as an efficient inhibitor of the Notch signaling, and therefore could constitute a promising therapeutic option for ovarian cancer patients.

## Additional files

Additional file 1: **Figure S1** Eugenol promotes cell death and inhibits cell growth of OC cells: **A.** OV2774 and SKOV3 cells were treated with different concentrations of eugenol for 24, 48 and 72 h, and cell growth was determined by the WST-1 assay (*n* = 3/group, mean +/− SD). **B.** Cells were treated as **A)** and cytotoxic dose response curves were determined by the WST-1 assay (n = 3/group, mean +/− SD). Results were processed and analyzed using the R-statistical software DRC package. (TIFF 712 kb)

Additional file 2: **Figure S2** Ectopic epression of Hes1 promotes invasion and resistance to cisplatin-induced apoptosis. **A.** Distribution and alteration frequency of Hes1, Hey1, Notch1, Notch2 and Notch3 in breast, ovarian, bladder, head and neck (HNCC) and clear cell renal cell carcinoma (ccRCC) cancer types. The data were obtained from cBioportal for Cancer Genomics (https://www.cbioportal.org/) and processed by the R- statistical software using oncoPrint function implemented in the ComplexHeatmp package. **B.** immunofluorescence staining of Hes1 in parental and sphere cultures after cisplatin treatment as indicated. Bar graphs indicate the positive staining of Hes1 in GFP^**+**^ and GFP^**−**^ cells (** *P* 0.01). **C**. Representative immunostaining images of cleaved caspase-3 (red color) in GFP^**+**^ and GFP^**−**^ cells. (** *p* 0.01). **D.** Transwell invasion assay of the indicated cells (** *p* 0.01). (TIFF 1359 kb)

Additional file 3: **Figure S3** Characterization of CD44^high^ and ALDH^high^ OC cells for stemness and self-renewal capacities. **A.** Characterization of Hes1-GFP^+^ cell fractions of OV2774 and SKOV3 cells, isolated based on their CD44 and ALDH positive cell expression levels. **B.** Confirmation of CD44 and ALDH1 expression from sorted cells shown as Hes1GFP^**+/−**^ by Western blotting. **C.** Hes1-GFP^**+**^ cells were grown in suspension and allowed to form spheres. After 10 days of culture, cells were sorted for CD44^**high**^/CD44^**low**^, ALDH^**high**^/ALDH^**low**^ and limiting dilution assay was performed by plating cells to ultra-low attachment 6-well plates. **D.** Morphological differences in spheres in both CD44^**high**^**/**^**low**^ and ALDH^**high**^**/**^**low**^ populations presented as representative photomicrographs. **E and F.** The sphere forming assay was performed with sorted CD44^**high**^, ALDH^**high**^ and CD44^**low**^/ALDH^**low**^. The number of spheres were expressed as mean+/−SEM, *n* = 3. (TIFF 903 kb)

Additional file 4: **Figure S4.** Side population analysis: **A.** Cells were treated with verapamil for 48 h, trypsinized and labelled with Hoechst 33342 dye and sorted for SP and NSP before and after verapamil treatment. **B**. The migration efficiency of CD44^high^ and ALDH^**high**^ cells presented as bar graph (mean+/−SEM, n = 3, * *p* 0.05). **C.** Correlation matrix between the drug resistance, CSC and pluripotency markers and Hes1. The color and width of the circle show the strength of the correlation between the variables. A full circle indicates the stronger correlation. An “r” values tables for each gene are appended below the “CORRPLOT”. The figure was drawn with the CORRPLOT package to create a CORRELOGRAM designed for R (http://r-bioconductor.org). (TIFF 2265 kb)

Additional file 5: **Figure S5.** Co-expression of Hes1, CD44 and ALDH in OC cells. **A.** Representative immunofluorescence staining of CD44 (green), ALDH (green) and Hes1 (red) and DAPI (4,6-diamidino-2-phenylindole) [blue] of OV2774 and SKOV3 cells. Images were captured using confocal microscope. **B.** Quantification of staining intensity (+ve vs –ve) of CD44, ALDH and Hes1showing weak to strong staining intensity in treated and untreated cells (**p 0.01, *** *p* 0.001). (TIFF 4893 kb)

Additional file 6: **Figure S6.** Eugenol sensitizes ovarian cancer cells to cisplatin in vivo: H&E staining as well as immunostaining of OV2774 xenograft tumor tissues using antibodies against the indicated proteins after treatment with eugenol and cisplatin alone and combination of both drugs. The graph bar represents quantification of the Ki-67 immunofluorescence. (n = 3, mean +/− SD; Students *t*-Test;*p 0.05, ** *p* 0.01, *** *p* 0.001). (TIFF 3537 kb)

Additional file 7: Materials and methods (DOCX 21 kb)

Additional file 8: **Table S1.** Antibodies used for FACS analysis. **Table S2A.** Relative inhibitory effects of cisplatin, eugenol alone and combination of both drugss for OV2774 and SKOV3 ovarian cancer cells: Cells were exposed to a range of cisplatin and eugenol concentrations for 72 h. Values indicate the relative inhibitory effects as means of +/− standard errors (SE) of the mean for at least 3 separate experiments. **Table S2B.** Sequential drug delivery of cisplatin and eugenol in OV2774 and SKOV3 human ovarian cancer cell lines. For details please refer to Materials and methods. CI (Combination Index), a quantitative method to measure the degree of drug interaction was calculated using CompuSyn Software. Note: The CI value < 1 indicate synergy; CI = 1 indicates additive, and CI > 1 indicates antagonism. **Table S3.** Antibodies used for immunofluorescence. **Table S4.** Antibodies used for immunoblotting. **Table S5.** Primers set used for qRT-PCR (DOCX 76 kb)

## Data Availability

The data generated, used and analyzed in the current study are available from the corresponding author in response to reasonable request.

## Abbreviations

CSCs: Cancer stem cells
OC: Ovarian cancer
OCSCs: Ovarian cancer stem cells
