# Supplementary figures and images for: Sequential combination of cisplatin with eugenol targets ovarian cancer stem cells through the Notch-Hes1 signalling pathway
Source: J Exp Clin Cancer Res. 2019 Aug 30;38:382. doi: 10.1186/s13046-019-1360-3 (PMC6716935; doi:10.1186/s13046-019-1360-3)

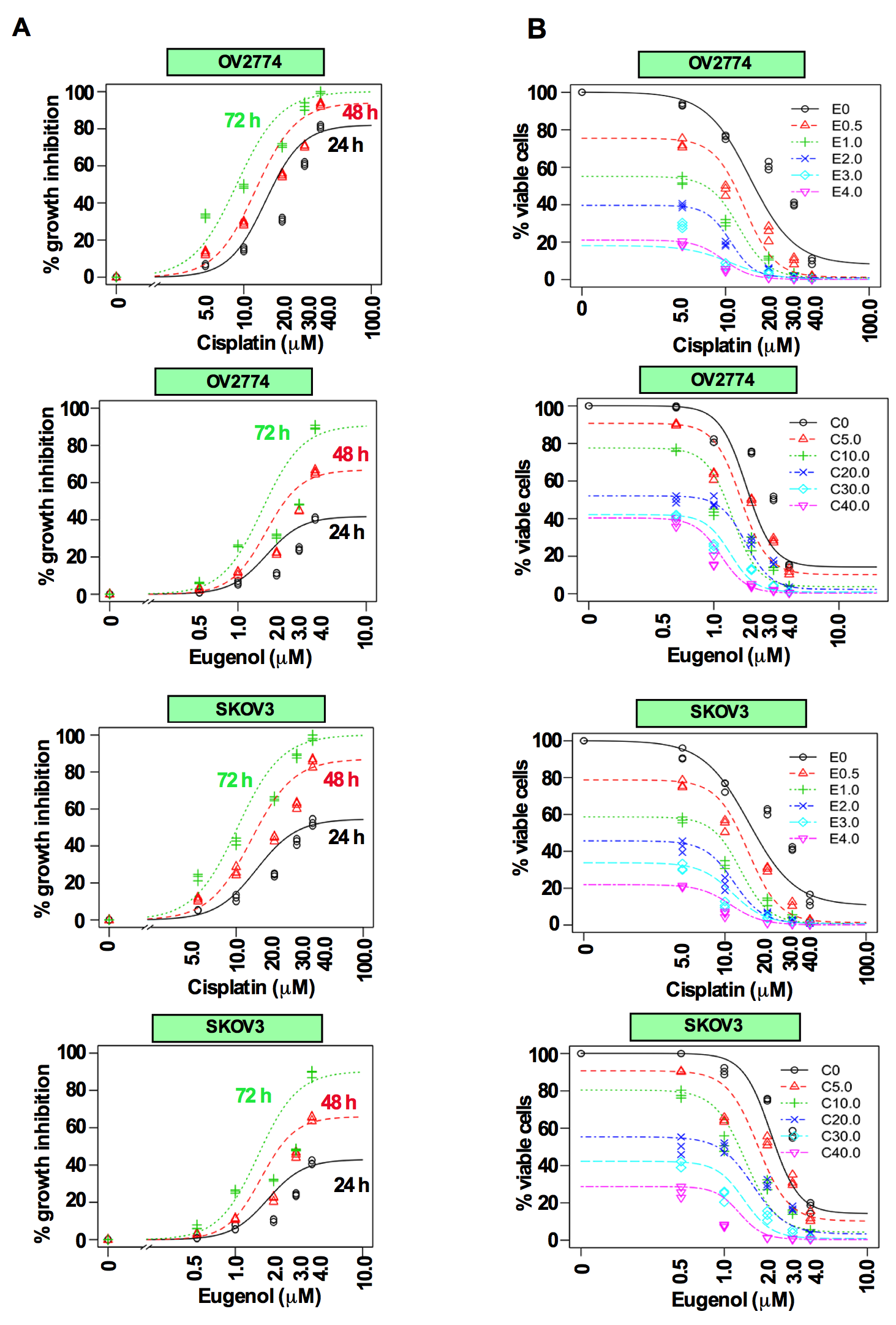

Supplement: Supplementary file 1 — Figure S1 Eugenol promotes cell death and inhibits cell growth of OC cells: A. OV2774 and SKOV3 cells were treated with different concentrations of eugenol for 24, 48 and 72 h, and cell growth was determined by the WST-1 assay (n = 3/group, mean +/− SD). B. Cells were treated as A) and cytotoxic dose response curves were determined by the WST-1 assay (n = 3/group, mean +/− SD). Results were processed and analyzed using the R-statistical software DRC package. (TIFF 712 kb) [file 13046_2019_1360_MOESM1_ESM.tiff]

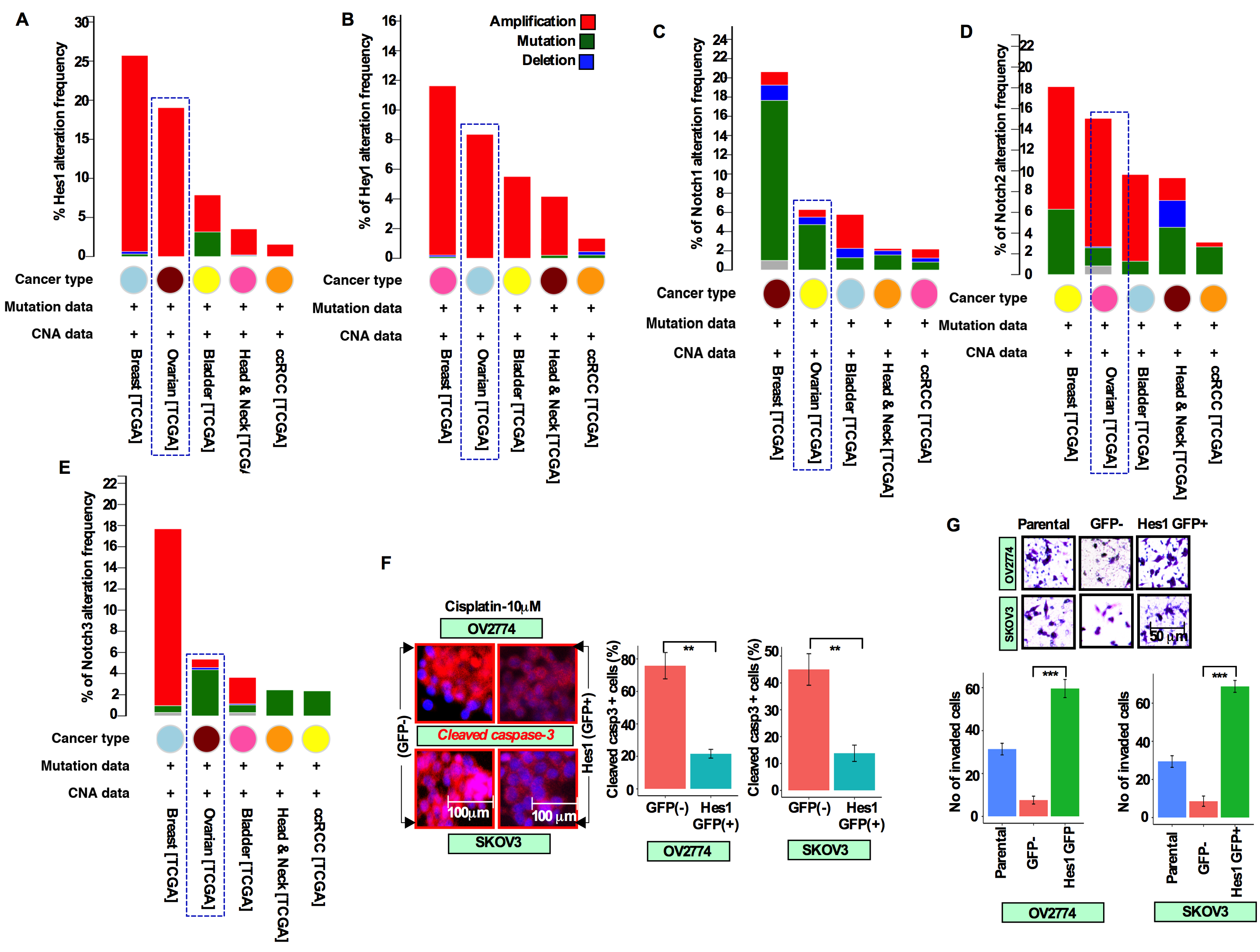

Supplement: Supplementary file 2 — Figure S2 Ectopic epression of Hes1 promotes invasion and resistance to cisplatin-induced apoptosis. A. Distribution and alteration frequency of Hes1, Hey1, Notch1, Notch2 and Notch3 in breast, ovarian, bladder, head and neck (HNCC) and clear cell renal cell carcinoma (ccRCC) cancer types. The data were obtained from cBioportal for Cancer Genomics (https://www.cbioportal.org/) and processed by the R- statistical software using oncoPrint function implemented in the ComplexHeatmp package. B. immunofluorescence staining of Hes1 in parental and sphere cultures after cisplatin treatment as indicated. Bar graphs indicate the positive staining of Hes1 in GFP+ and GFP− cells (** P 0.01). C. Representative immunostaining images of cleaved caspase-3 (red color) in GFP+ and GFP− cells. (** p 0.01). D. Transwell invasion assay of the indicated cells (** p 0.01). (TIFF 1359 kb) [file 13046_2019_1360_MOESM2_ESM.tiff]

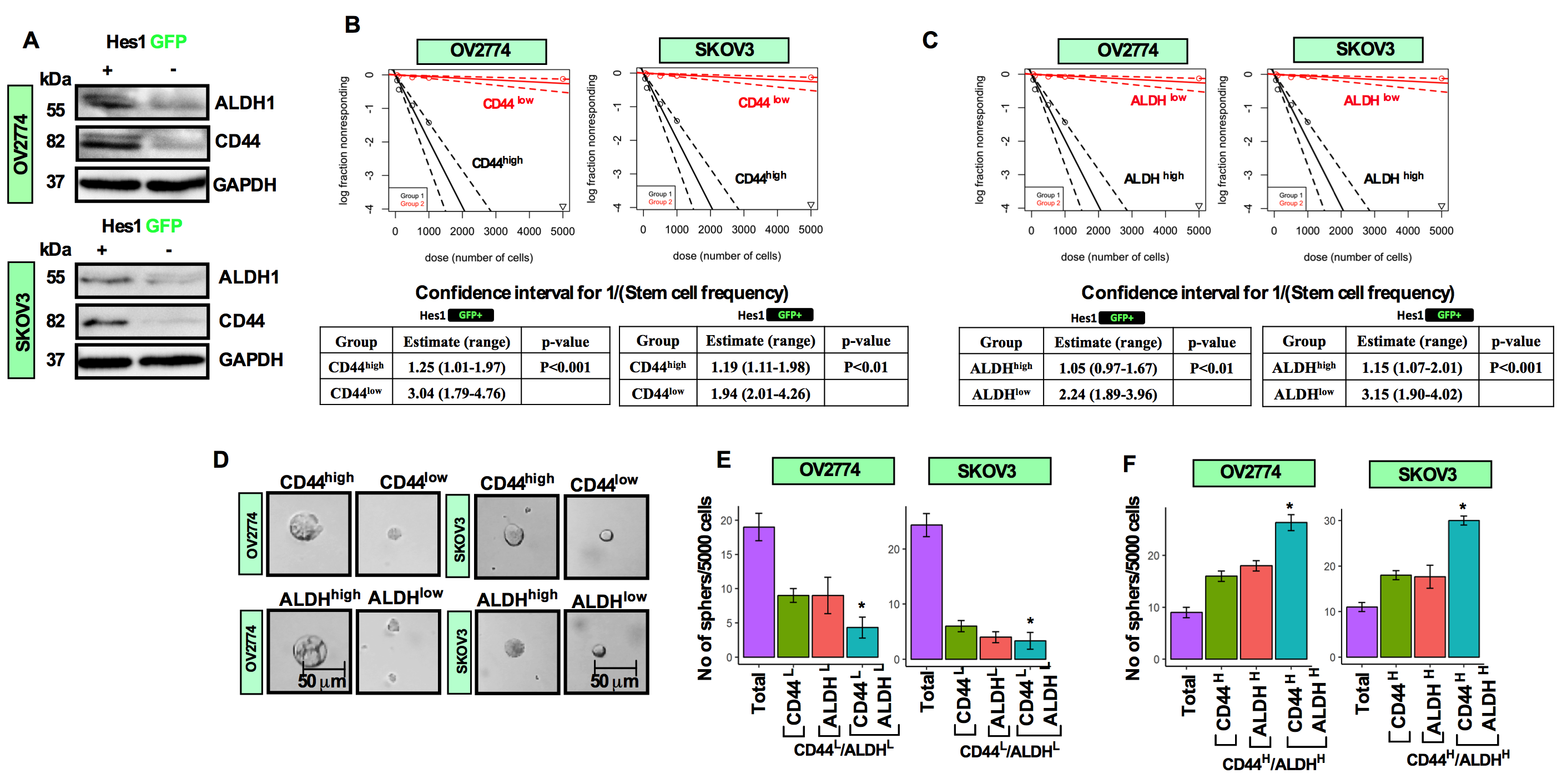

Supplement: Supplementary file 3 — Figure S3 Characterization of CD44high and ALDHhigh OC cells for stemness and self-renewal capacities. A. Characterization of Hes1-GFP+ cell fractions of OV2774 and SKOV3 cells, isolated based on their CD44 and ALDH positive cell expression levels. B. Confirmation of CD44 and ALDH1 expression from sorted cells shown as Hes1GFP+/− by Western blotting. C. Hes1-GFP+ cells were grown in suspension and allowed to form spheres. After 10 days of culture, cells were sorted for CD44high/CD44low, ALDHhigh/ALDHlow and limiting dilution assay was performed by plating cells to ultra-low attachment 6-well plates. D. Morphological differences in spheres in both CD44high/low and ALDHhigh/low populations presented as representative photomicrographs. E and F. The sphere forming assay was performed with sorted CD44high, ALDHhigh and CD44low/ALDHlow. The number of spheres were expressed as mean+/−SEM, n = 3. (TIFF 903 kb) [file 13046_2019_1360_MOESM3_ESM.tiff]

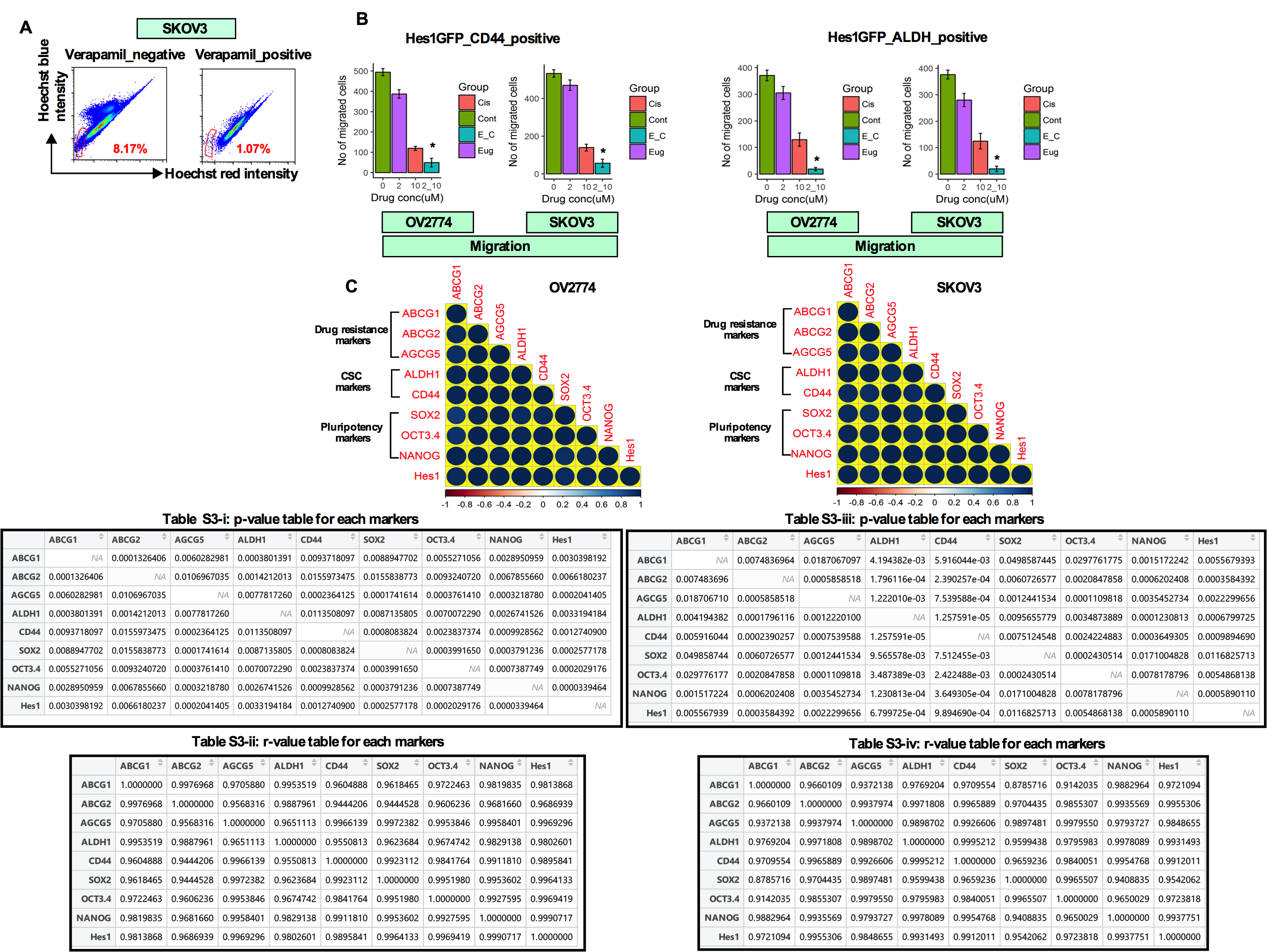

Supplement: Supplementary file 4 — Figure S4. Side population analysis: A. Cells were treated with verapamil for 48 h, trypsinized and labelled with Hoechst 33342 dye and sorted for SP and NSP before and after verapamil treatment. B. The migration efficiency of CD44high and ALDHhigh cells presented as bar graph (mean+/−SEM, n = 3, * p 0.05). C. Correlation matrix between the drug resistance, CSC and pluripotency markers and Hes1. The color and width of the circle show the strength of the correlation between the variables. A full circle indicates the stronger correlation. An “r” values tables for each gene are appended below the “CORRPLOT”. The figure was drawn with the CORRPLOT package to create a CORRELOGRAM designed for R (http://r-bioconductor.org). (TIFF 2265 kb) [file 13046_2019_1360_MOESM4_ESM.tiff]

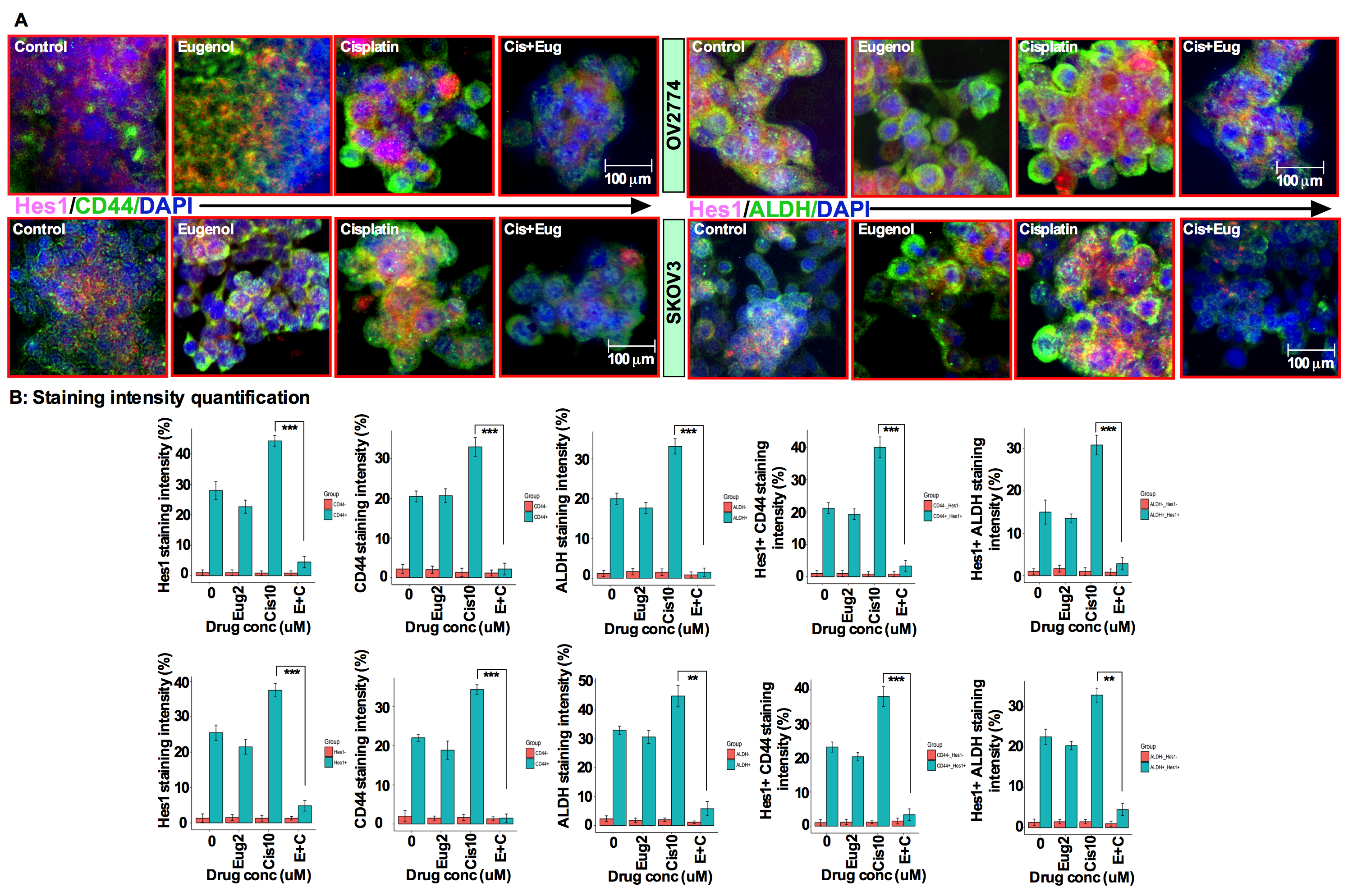

Supplement: Supplementary file 5 — Figure S5. Co-expression of Hes1, CD44 and ALDH in OC cells. A. Representative immunofluorescence staining of CD44 (green), ALDH (green) and Hes1 (red) and DAPI (4,6-diamidino-2-phenylindole) [blue] of OV2774 and SKOV3 cells. Images were captured using confocal microscope. B. Quantification of staining intensity (+ve vs –ve) of CD44, ALDH and Hes1showing weak to strong staining intensity in treated and untreated cells (**p 0.01, *** p 0.001). (TIFF 4893 kb) [file 13046_2019_1360_MOESM5_ESM.tiff]

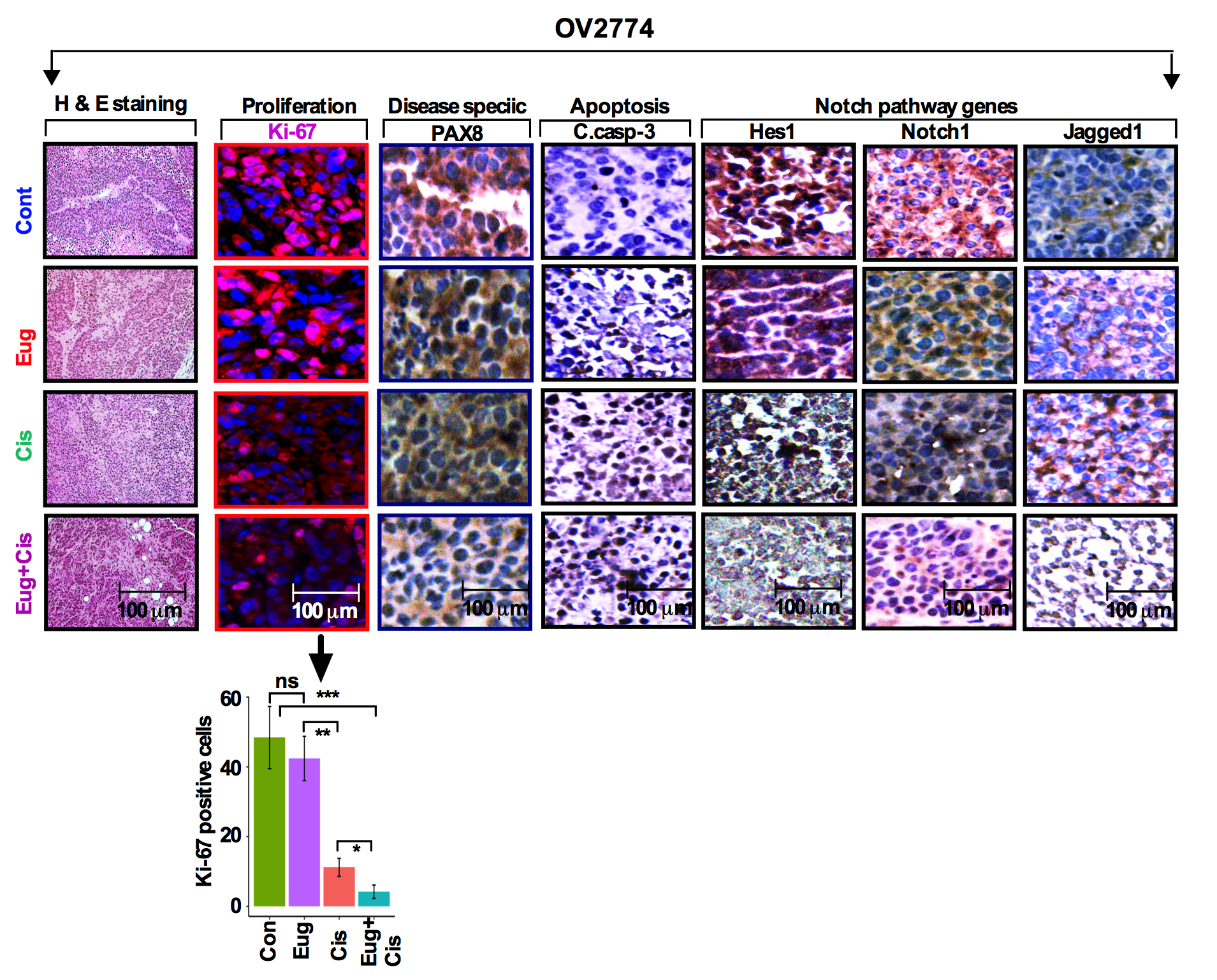

Supplement: Supplementary file 6 — Figure S6. Eugenol sensitizes ovarian cancer cells to cisplatin in vivo: H&E staining as well as immunostaining of OV2774 xenograft tumor tissues using antibodies against the indicated proteins after treatment with eugenol and cisplatin alone and combination of both drugs. The graph bar represents quantification of the Ki-67 immunofluorescence. (n = 3, mean +/− SD; Students t-Test;*p 0.05, ** p 0.01, *** p 0.001). (TIFF 3537 kb) [file 13046_2019_1360_MOESM6_ESM.tiff]
