# Supplementary material for: Sequential combination of cisplatin with eugenol targets ovarian cancer stem cells through the Notch-Hes1 signalling pathway
Source: J Exp Clin Cancer Res. 2019 Aug 30;38:382. doi: 10.1186/s13046-019-1360-3 (PMC6716935; doi:10.1186/s13046-019-1360-3)
Supplement: Supplementary file 7 — Materials and methods (DOCX 21 kb) [file 13046_2019_1360_MOESM7_ESM.docx]

**Supplementary Materials and methods:**

**Cell growth and cytotoxicity and apoptosis assays**

Cells in 96 well plates were treated with cisplatin (0-40 μM), eugenol (0-4 μM). Cell proliferation and cytotoxicity were analyzed by the WST-1 assay. For combination treatment and interaction of different concentrations of drugs, we used the methods described by Chou et al. Briefly OV2774 and SKOV3 cells were treated with either cisplatin for 24 hrs before or after eugenol treatment or vice versa. Treated cells were incubated for 72 hrs and analyzed by the WST-1 assay. Synergism, additive and antagonism in different combinations were calculated and quantitated by combination index (CI), where CI value =1 indicates that the response is additive, CI<1 indicates synergistic and CI>1 indicate antagonistic. Drug interaction and isobologram were analyzed using CompuSyn software (ComboSyn Inc).

**Annexin-V flow cytometry**

Upon treatment, cells were trypsinized, washed with PBS, and 5 μl of Annexin V-FITC (Invitrogen) and 5 μl of propidium iodide (PI) and incubated for 15 minutes at room temperature (25^0^ C). Finally, 200 μl of 1x binding buffer was added and run by flow cytometer (NovoCyte, USA) and analyzed the data by NovoExpress software.

**Flow cytometry of CD44 and ALDH**

Cells were stained with anti-CD44-APC antibody (1:20; BD Bioscience, cat#559942) at room temperature for 45 mins. Hes1-positive cells were isolated based on the expression of CD44 and ALDH. After sorting, isolated cells were washed twice with sterile PBS, centrifuged for 5 minutes before each wash cycle for in vitro assays.

**SP and NSP cell fraction isolation and purification**

SP and NSP cells were isolated and identified using previously described methods (1). Cells were resuspended in DMEM containing 2% FBS and labeled with H33342 (Sigma, St Louis, ) at a concentration of 2.5 μg/ml for 60 minutes in 37^0^ C or with 225μM of verapamil hydrochloride (Sigma, USA). Cells were centrifuged and resuspended in PBS with 2% FBS. The cells were then passed through 40-μm mesh filter, counter stained with 10 μg/ml propidium iodide. Cells were analyzed using (NovoCyte, Novo Express software) or sorted using FACSAria cell sorter (BD Bioscience, USA). and analyzed with a FACSDiva software. Following isolation, SP- and NSP cells were used for *in vitro* experiments.

**Immunofluorescence and image analysis**

The immunofluorescence staining was performed as described previously (2). In short, cells were cultured on glass cover slip, washed with PBS and fixed in ice cold methanol. Permeabilized with 0.1% Triton X for 3 minutes, blocked with 5% BSA for thirty minutes. Afterwards incubated with primary antibodies [1;100, Sigma, USA] overnight at 4^0^ C. Finally, washed with PBS and blocked secondary antibody and mounted the cover slips with mounting medium (Vecta Shield, USA) with DAPI (4,6-diamidino-2-phenylindole). Analyzed and photographed by fluorescence microscope to measure the CD44 and Hes1+ cells. Quantification of CD44+ and Hes1+ cells were performed using ImageJ (NCBI) freeware. CD44+ and Hes1+ and CD44+/Hes1+ were analyzed by choosing positive cells/6 randomly selected areas for each cover slip (Scale is 100 μm). The following parameters were used to defined weak/negative, medium and strong staining intensity: weak/negative was defined as “0”, medium as “2” and strong as “4”. The staining intensity was converted into percentage of whole cover slip area and presented as bar graph using R-statistical software package ([www.https://cran-r.project.org/web/packages/ggplot2)](http://www.https://cran-r.project.org/web/packages/ggplot2) “ggplot2”. Complete list of used antibodies can be found in the Supplementary Table S3.

**Western blotting**

Western blotting was performed as described previously (2). Complete list of used antibodies can be found in the Supplementary Table S4.

**Quantitative RT-PCR**

Total RNA was extraction and reverse transcription were performed using the method described previously (2). Supplementary Table S4 lists all the primers used in this study. The results were normalized with GAPDH. List of all primers can be found in Supplementary Table S5.

**Invasion assay**

Invasion assay was performed as described previously (2). Briefly, SKOV3 and OV2774 cells were treated with cisplatin, eugenol or combination of both for 72 hrs. Invaded cells were stained with 1% crystal violet dye. The number of positive stained were counted and photographed using phase contrast microscope. Ten (10) randomly selected fields were counted and data were analyzed and presented as bar graph.

**Aldefluor assay and sorting of ALDH-positive cell population by Flow cytometry**

Cells were suspended in Aldefluor assay (Stem cell technologies, Vancouver, BC, Canada) buffer containing ALDH substrate (Bodipy-aminoacetaldehyde) and incubated for 45 minutes at 37° C. As control, cells were suspended in buffer containing Aldefluor substrate in the presence of specific ALDH enzyme inhibitor diethylaminobenzaldehyde (DEAB). Desired ALDH-positive cells populations were isolated using the FACSAria flow cytometer (BD Bioscience, San Diego, USA) and the data were analyzed by the FACS DIVA software (BD Bioscience). After sorting, cells were washed twice with sterile PBS and were centrifuged for 5 minutes before each wash cycle for in vitro assays.

**Limiting dilution assay**

Sorted GFP+ cells were diluted into ultra-low attachment 6-well plates with 1ml of DMEM/F12 (1:1) per well, supplemented with 2% B27 supplements (Invitrogen), 20 ng/ml of epidermal growth factor (EGF, Sigma), 20 ng/ml of basic fibroblast growth factor (bFGF, Sigma), 2ug/ml hydrocortisone (Sigma), 2 μg/ml insulin (Sigma), 2 μg/ml heparin (Stem Cell Technologies) for 20 days. Tumorspheres were monitored every day and counted under phase contrast microscope. Data was analyzed using Extreme Limited Dilution Analysis (ELDA) by “R- statistical software and STATMOD packages ([www.https://cran-r.project.org/web/packages/statmod](http://www.https://cran-r.project.org/web/packages/statmod)” to determine the stem cell frequencies.

**MitoSOX Red mitochondrial ROS activity measurement**

Treated cells were washed with PBS, trypsinized, and mitochondrial ROS was measured by incubating cells with 5μM MitoSOX Red (Life Technologies, USA), incubated in 37^0^ C for 30 mins, washed with PBS, resuspended with 100 μL PBS and immediately analyzed by NovoCyte flow cytometry.

**Soft agar colony formation assay**

Cells were treated with drugs and harvested and washed with serum free medium and suspended in 4 ml of defined medium with 0.3% agarose (A9045, Sigma, MD, USA). The mixture of agarose and cells was plated in 6-well plate containing a base layer of agarose of 0.5% (v/v). Cultures were incubated at 37^0^ C in a humidified incubator. Medium were changed every 3 days and cultures were continued for 21 days. Colonies were counted 1 week after initial plating. Colony size, number of colonies and growth of colonies were recorded. Results show mean of three separate experiments.

**References for Supplemental data**

1. Goodel MA., Brose K., Paradis G., Conner AS., Mulligan RC. Isolation and functional properties of murine hematopoetic stem cells that are replicating in vivo. J Exp Med 1996;**183**: 1797-1806.
2. Islam SS., Al-Sharif I., Sultan A., Al-Mazrou A., Remmel A., Aboussekhra A. Eugenol potentiates cisplatin anti-cancer activity through inhibition of ALDH-positive breast cancer stem cells and the NF-kB signaling pathway. Mol Carcinog 2017;1-14, doi:10.1002/mc.22758.
