# Supplementary material for: Sequential combination of cisplatin with eugenol targets ovarian cancer stem cells through the Notch-Hes1 signalling pathway
Source: J Exp Clin Cancer Res. 2019 Aug 30;38:382. doi: 10.1186/s13046-019-1360-3 (PMC6716935; doi:10.1186/s13046-019-1360-3)
Supplement: Supplementary file 8 — Table S1. Antibodies used for FACS analysis. Table S2A. Relative inhibitory effects of cisplatin, eugenol alone and combination of both drugss for OV2774 and SKOV3 ovarian cancer cells: Cells were exposed to a range of cisplatin and eugenol concentrations for 72 h. Values indicate the relative inhibitory effects as means of +/− standard errors (SE) of the mean for at least 3 separate experiments. Table S2B. Sequential drug delivery of cisplatin and eugenol in OV2774 and SKOV3 human ovarian cancer cell lines. For details please refer to Materials and methods. CI (Combination Index), a quantitative method to measure the degree of drug interaction was calculated using CompuSyn Software. Note: The CI value < 1 indicate synergy; CI = 1 indicates additive, and CI > 1 indicates antagonism. Table S3. Antibodies used for immunofluorescence. Table S4. Antibodies used for immunoblotting. Table S5. Primers set used for qRT-PCR (DOCX 76 kb) [file 13046_2019_1360_MOESM8_ESM.docx]

Additional file 8

**Table S1:** Antibodies used for FACS analysis

| Anti-human CD44-APC (clone) | BD Bioscience | 55-9942 |
| --- | --- | --- |
| Anti-human-Hes1-FITC (clone) | Invitrogen | 11988 |
| Anti-human cleaved caspace 3 (clone) | Cell Signaling | 9664 |

**Table S2A:** Relative inhibitory effects of cisplatin, eugenol alone and combination of both drugss for OV2774 and SKOV3 ovarian cancer cells: Cells were exposed to a range of cisplatin and eugenol concentrations for 72hrs. Values indicate the relative inhibitory effects as means of +/- standard errors (SE) of the mean for at least 3 separate experiments.

**OV2774 cell line**

**SKOV3 cell line**

**Table S2B:** Sequential drug delivery of cisplatin and eugenol in OV2774 and SKOV3 human ovarian cancer cell lines. For details please refer to Materials and methods. CI (Combination Index), a quantitative method to measure the degree of drug interaction was calculated using CompuSyn Software. Note: The CI value <1 indicate synergy; CI=1 indicates additive, and CI>1 indicates antagonism.

**Table S3:** Antibodies used for immunofluorescence

| Anti-human Hes1 | Invitrogen | 11988 | 1:300 |
| --- | --- | --- | --- |
| Anti-human cleaved caspase 3 | Cell Signaling | 9664 | 1:300 |
| Anti-human CD31 | BD Bioscience | 553369 | 1:200 |
| Anti-human PAX8 | Abcam | ab63490 | 1:100 |
| Anti-human Ki-67 | Abcam | ab15580 | 1:200 |
| Anti-human Notch1 | EMD Millipore | 07-1232 | 1:200 |
| Anti-human Jagged1 | Thermo Fischer | PA5-46970 | 1:200 |
| Antihuman CD44 | Sigma | HPA005785 | 1:300 |
| Anti-human ALDH1 | Sigma | SAB140342 | 1:300 |

**Table S4:** Antibodies used for immunoblotting

| Cleaved caspase-3 | Cell Signaling | 9664 | 1:300 |
| --- | --- | --- | --- |
| Cleaved-PARP | Cell Signaling | 9541 | 1:300 |
| Hes1 | Cell Signaling | 11988 | 1:300 |
| Notch1 | EMD Millipore | 07-1232 | 1:500 |
| Hey1 | Sigma | SAB2106265 | 1:200 |
| Jagged1 | Thermo Fischer | PA5-46970 | 1:200 |
| c-Myc | BD Pharmingen | 551101 | 1:200 |
| Phospho-AKT | Cell Signaling | 9275 | 1:400 |
| AKT | Cell Signaling | 9275 | 1:400 |
| CD44 | Sigma | HPA005785 | 1:300 |
| ALDH1 | Sigma | SAB140342 | 1:300 |
| Presenillin | Abcam | ab106351 | 1:400 |
| Nicastrin | Abcam | ab189125 | 1:400 |
| APH1-alpha | Abcam | ab111992 | 1:500 |
| PEN2 | Abcam | ab154830 | 1:400 |
| GAPDH | Santa Cruz | sc25778 | 1:200 |

**Table S5: Primers set used for qRT-PCR**

| **Genes** | **Forward** | **Reverse** |
| --- | --- | --- |
| Hes1 | ACA CGA CAC CGG ATA AAC CAA | CGA GTG CGC ACC TCG GTA |
| ABCG1 | GAC CGG ACA TCC CAG TGC TT | TGT GCT CGG AGA CAC TGA AC |
| ABCG2 | TGG CTG TCA TGG CTT CAG TA | GCC ACG TGA TTC TTC CAC AA |
| ABCG5 | CAC AAA AGG CCA TTC AGG CT | GCT GAG GAA TCC ACC CAA TCT |
| EpCam | GAA TGG CAA AGT ATG AGA AGG CTG A | TCC CAC GCA CAC ACA TTT GTA A |
| CD49f | ACC CAG ATA TTG CAG TTG GA | TTC GAT CAA GGT CCA TGT TT |
| ALDH1 | TGT TAG CTG ATG CCG ACT TG | TTC TTA GCC CGC TCA ACA CT |
| CD44 | TGA ATA TAA CCT GCC GCT TTG | GTC ATA CTG GGA GGT GTT GGA |
| Sox2 | GGT TTC CAG TTC TTG CAC GCT GTT | TTG CAA GGT AGG AAG CCA AGA AGC |
| Oct3/4 | GTA CTC CTC GGT CCC TTT CC | CAA AAA CCC TGG CAC AAA CT |
| Nanog | TTC CTT CCT CCA TGG ATC TG | TCT GCT GGA GGC TGA GGT TAT |
| GAPDH | CGA CCA CTT TGT CAA GCT CA | AGG GGT CTA CAT GGC AAC TG |
